# Network-Based and Machine-Learning Approaches Identify Diagnostic and Prognostic Models for EMT-Type Gastric Tumors

**DOI:** 10.3390/genes14030750

**Published:** 2023-03-19

**Authors:** Mehdi Sadeghi, Mohammad Reza Karimi, Amir Hossein Karimi, Nafiseh Ghorbanpour Farshbaf, Abolfazl Barzegar, Ulf Schmitz

**Affiliations:** 1 Department of Cell & Molecular Biology, Semnan University, Semnan 3513119111, Iran; 2 Research Institute for Fundamental Science, University of Tabriz, Tabriz 5166616471, Iran; 3 Department of Biology, Faculty of Natural Science, University of Tabriz, Tabriz 5166616471, Iran; 4 Department of Molecular & Cell Biology, James Cook University, Townsville, QLD 4811, Australia; 5 Centre for Tropical Bioinformatics and Molecular Biology, Australian Institute of Tropical Health and Medicine, James Cook University, Cairns, QLD 4878, Australia

**Keywords:** gastric cancer, epithelial-mesenchymal transition, EMT subtype, precision medicine, WGCNA, machine learning, microRNA, motif analysis

## Abstract

The microsatellite stable/epithelial-mesenchymal transition (MSS/EMT) subtype of gastric cancer represents a highly aggressive class of tumors associated with low rates of survival and considerably high probabilities of recurrence. In the era of precision medicine, the accurate and prompt diagnosis of tumors of this subtype is of vital importance. In this study, we used Weighted Gene Co-expression Network Analysis (WGCNA) to identify a differentially expressed co-expression module of mRNAs in EMT-type gastric tumors. Using network analysis and linear discriminant analysis, we identified mRNA motifs and microRNA-based models with strong prognostic and diagnostic relevance: three models comprised of (i) the microRNAs miR-199a-5p and miR-141-3p, (ii) EVC/EVC2/GLI3, and (iii) PDE2A/GUCY1A1/GUCY1B1 gene expression profiles distinguish EMT-type tumors from other gastric tumors with high accuracy (Area Under the Receiver Operating Characteristic Curve (AUC) = 0.995, AUC = 0.9742, and AUC = 0.9717; respectively). Additionally, the DMD/ITGA1/CAV1 motif was identified as the top motif with consistent relevance to prognosis (hazard ratio > 3). Molecular functions of the members of the identified models highlight the central roles of MAPK, Hh, and cGMP/cAMP signaling in the pathology of the EMT subtype of gastric cancer and underscore their potential utility in precision therapeutic approaches.

## 1. Introduction

Gastric cancer (GC) is one of the most common malignancies with extreme inter- and intra-tumoral heterogeneity [1,2]. With more than a million new cases each year and approximately 769,000 deaths in 2020, it comprises one of the leading causes of cancer-related deaths worldwide [3]. Despite its substantial burden, little progress has been made regarding the development of effective therapeutic interventions for GC patients [4]. This reflects the inability of the conventional one-size-fits-all diagnostic/therapeutic approaches for combatting such a heterogeneous disease. 

Fortunately, in recent decades, various classifications with either histologic [5] or molecular [6] bases have been developed for this malignancy. These classification systems guide the development of disease management strategies that are tailor-made for specific subtypes of GC. In comparison with histologic classifications, molecular classifications display a wider association with tumor heterogeneity and patient prognosis, suggesting their broader utility in the clinical setting [7]. One of the major molecular classifications of stomach cancer was developed based on the mRNA expression data of gastric tumors almost a decade ago by the Asian Cancer Research Group (ACRG) [8]. This classification stratifies gastric tumors into four subtypes, namely (i) microsatellite instability (MSI), (ii) microsatellite stable/epithelial-mesenchymal transition (MSS/EMT; EMT for short), (iii) microsatellite stable/TP53+, and (iv) microsatellite stable/TP53−. Among these, the EMT subtype is associated with significantly poorer overall survival and a higher chance of recurrence, possibly demanding a more aggressive treatment approach [8,9,10].

Despite the obvious benefits of tumor classifications, the substantial costs of the current experimental approaches required for patient stratification impede the clinical translation of these subtypes, underscoring the necessity of the development of practical biomarkers for disease/patient management [7]. Specifically, considering the aggressive nature of the EMT-type tumors, exploration of the molecular landscape of these tumors and the development of practical means for the stratification of patients into EMT and non-EMT cases is of substantial interest. In this line, Lee at el. [9] developed a NanoString-based 71-gene signature assay that can potentially be used for diagnostic/prognostic purposes in the clinical setting. Nevertheless, there is still room for reductions in the costs and availability of patient stratification approaches, and the underlying biology of the phenotypes observed in patients with EMT-type tumors remains elusive.

In this study, we established the EMT GC subtype, proposed by the ACRG, as the subtype with the most distinct transcriptomic landscape and moved on to identify some of the core elements involved in the pathology of this subtype through the combination of co-expression module discovery and motif extraction approaches. These elements were further explored in terms of their clinical utility, and the most potent candidates with diagnostic and prognostic relevance were identified and discussed. The pipeline designed for this study appears to be robust for the identification of central regulators of biological phenomena and can readily be employed in other similar contexts. Moreover, the top motifs identified represent potent candidates for further validation to be used as affordable means for the stratification of GC patients in the clinical setting. 

## 2. Materials and Methods

### 2.1. Datasets

We retrieved RNA-seq and miRNA-seq raw counts from treatment-naïve adenocarcinomas of The Cancer Genome Atlas-STomach ADenocarcinoma (TCGA-STAD) cohort (*n* = 316; only the samples that were not flagged as low quality were retrieved) using the Genomic Data Commons (GDC) data portal [11] and microarray data from the ACRG cohort (*n* = 300) and the Singapore cohort (*n* = 192) via the Gene Expression Omnibus (accession numbers GSE62254 and GSE15459). The clinical information for the analyzed samples is available in the Supplementary Table S1. The distribution of the clinical information within each subtype for all three cohorts is also presented in Table 1. Since not all of the 316 TCGA samples possessed all the required data categories for the different steps of the analysis (e.g., survival data, ACRG classification, etc.), for each specific step of the study, only the subset of the original cohort that included all data modalities relevant to that step was utilized. Tumors from all three cohorts have been previously classified into the four ACRG-based molecular subtypes [8]. The same classification was used in this study. This reduced the samples with classifications for the TCGA to a total of 167 samples (MSI = 37; EMT = 47; TP53+ = 42; TP53− = 41). In the ACRG cohort, three samples (#369, #533, and #542) were removed since they were identified as outliers based on the Principal Component Analysis (PCA) of the log2 transformed intensities (total: 297; EMT = 46; MSI = 68; TP53+ = 77; TP53− = 106). The RNA-seq data for gastric tumors and paired normal gastric tissues were also retrieved from GSE184336 for tumor vs normal comparisons.

### 2.2. Data Analysis and Visualization

R version 4.1.1 and Cytoscape version 3.9.0 were used for the statistical and network-based analysis of the data and visualization of the results. Differential gene expression analysis was carried out using the DESeq2 R package [12], which uses negative binomial generalized linear models for the identification of the differentially expressed genes. Venn diagrams were constructed using the VennDiagram package and PCA was carried out using the prcomp function in R.

### 2.3. Evaluation of ACRG Subtypes

Enrichment analysis of the TCGA tumor samples classified into the four distinct subtypes in comparison to the normal samples was carried out using the Hallmark gene sets of the Gene Set Enrichment Analysis (GSEA) desktop application version 4.1.0 [13]. GSEA is one of the most popular methods from the second generation of enrichment analysis techniques. This method ranks genes based on the correlation of their expression levels with the phenotype under investigation and calculates an enrichment score for each predefined gene set (in this case, the gene sets in the Hallmark collection of the GSEA) based on the aggregation of the members of these sets at the top or the bottom of the ranked list of genes. Identification of the top modules of the differentially expressed genes for each subtype was conducted using the greedy search algorithm of the jActiveModules plug-in in the Cytoscape [14].

### 2.4. Weighted Gene Co-Expression Network Analysis and Motif Identification

Co-expression modules are, in essence, clusters of genes that present a coordinated variation in their expression levels across samples, and they potentially represent groups of genes with related functions regulated by the same transcriptional program. The interpretation of these modules within specific biological contexts can reveal novel insights regarding how specific functions/phenotypes are regulated [15]. Here, the identification of co-expression modules was performed using the Weighted Gene Co-expression Network Analysis (WGCNA) algorithm [16]. WGCNA first constructs an adjacency matrix by applying a hard or soft thresholding procedure on the co-expression similarity measurements between each pair of genes and then utilizes a clustering approach for the identification of the co-expression modules. In this study, the co-expression module discovery was carried out with the following parameters: a signed topological overlap matrix was used, the minimum module size was set to 20, the optimum soft threshold was identified as 20 using the scale independence and mean connectivity plots, and the dendrogram cut height for module merging was set to 0.25. The significance of the modules was determined by taking the average of the −log10(adj. *p*-value) of the differential expression of their members in the EMT samples compared to the pooled samples of the other subtypes (Wald test; corrected for multiple hypothesis testing by the Benjamini–Hochberg method). 

Motifs in protein–protein interaction (PPI) networks are small subgraphs that occur much more often than is expected by chance. Alterations in the activity and expression levels of these regulatory units are a common observation in pathological states such as cancer [17]. In this context, the identified top module was further queried for biologically relevant regulatory subunits through the utilization of motif identification approaches. The PPI data were retrieved from the STRING database version 11.5 [18], and the NetMatchStar plug-in in the Cytoscape [19] was used to identify triangle motifs with three nodes and three edges. The choice of the triangle motifs was based on the high frequency with which they are observed in the biological systems and the fact that many larger motifs are comprised of multiple triangle motifs [20].

A modified version of the multi-objective scoring function used in [21,22] was used for motif scoring:

$$S_{ij}=\frac{W_{1j}}{2}\times \frac{{\left(ND\right)}_{i}}{\max\left(ND\right)}+\frac{W_{1j}}{2}\times \frac{{\left(BC\right)}_{i}}{\max\left(BC\right)}+W_{2j}\times \frac{{\left(DP\right)}_{i}}{\max\left(DP\right)}+W_{3j}\times \frac{{\left(AUC\right)}_{i}}{\max\left(AUC\right)}+W_{4j}\times \frac{{\left(\left|LFC\right|\right)}_{i}}{\max\left(\left|LFC\right|\right)},$$

where *W* stands for the weight, *i* is any given motif, *j* is any one of the weighting scenarios (all of the 13 utilized weighting scenarios are available in the Supplementary Table S2), *ND* is the mean of the node degree of each of the motif members, *BC* is the mean betweenness centrality, *DP* is the number of the nodes in a given motif that are members of the pathways in the cancer KEGG pathway (hsa05200), *AUC* is the mean area under the *ROC* curve, and the *LFC* is the mean absolute log2 fold change of the expression of the nodes in a motif in the *EMT* subtype in comparison to the pooled samples of the other subtypes. The ‘max (parameter)’ denotes the maximum value of each parameter achieved by a motif.

### 2.5. Assessment of Diagnostic and Prognostic Values of the RNAs

Survival analysis was performed using the survival and survminer packages in R. The TCGA RNA-seq data for 288 solid tumor samples with appropriate clinical information based on the criteria used by Anaya [23] were subjected to Variance Stabilizing Transformation (VST), and the ACRG microarray data were Robust Multichip Average (RMA)-normalized prior to the survival analysis.

The top and bottom 40% of the samples (based on the expression of the gene under investigation) were used as the high-expression and low-expression groups, respectively. As for the motifs, the intersection of the samples in the top/bottom 40% based on the expression of each motif member was used to form the high-expression and low-expression groups. The age and sex of the patients were used as covariates in the Cox regression analysis in order to account for their possible confounding effects. Due to the inclusion of samples that exhibited concordant high/low expression of all of the motif members in each analysis, a varying number of samples were analyzed for each motif. Considering this, only motifs with at least 30 samples in each group (high- and low-expression groups) and a total of at least 100 samples were selected for further examination. Among these, we specifically looked for motifs that were consistently present among the top five motifs of both cohorts (based on their Hazard Ratio [HR]). 

The glm built-in function in R was used for the logistic regression analysis. Since quantile normalization was found to be an excellent method for making the microarray and RNA-seq data comparable for machine learning applications [24], the raw counts and intensities for TCGA and ACRG samples were pooled, log2 transformed, and quantile normalized prior to logistic regression analysis. After normalization, the TCGA and ACRG samples were again separated, and the regression models for discrimination between tumor subtypes were first fitted to the TCGA data and then validated on the ACRG data. To assess the robustness of the models, their performance on the independently quantile normalized data of the samples from the Singapore cohort was also evaluated. The ability of the motifs to distinguish tumors from normal samples was also assessed by fitting a model to the TCGA RNA-seq data for both STAD solid tumors (*n* = 316) and the available adjacent normal tissue samples from the gastric cancer patients in the TCGA-STAD cohort (*n* = 30; cases for which adjacent normal tissue samples were available are distinguished with bold script in the Supplementary Table S1) after VST normalization. The same method was also applied to the GSE184336 dataset (with 70% of the samples as the training set and the remaining samples as the validation set) for independent validation of the capacity of the motifs for discrimination between normal and tumor samples. 

Multi-candidate miRNA combinations capable of discriminating EMT-type tumors from other subtypes were identified using the linear discriminant analysis (LDA) with leave-one-out cross-validation, using the method described in [25]. Eighty percent of the samples were allocated to the training set for this analysis and the remaining samples were used for validation. The validated mRNA targets of the differentially expressed miRNAs were obtained using the multiMiR library in R [26].

### 2.6. MiRNA-mRNA Network Construction

The miRNA-mRNA network was constructed in R using the PPI interaction information from STRING and the validated miRNA-target interactions obtained from multiMiR. Twenty-three centrality measures were calculated for the network using the igraph and centiserve [27,28] packages in R. PCA was used to identify the most suitable centrality measure among these 23 centrality measures based on the structure of the network, using the method described in Ashtiani et al. [29]. The final network was visualized using Cytoscape.

## 3. Results

### 3.1. EMT-Type Gastric Cancer Displays a Distinct Transcriptional Profile

In order to assess the transcriptional rewiring of the tumors in different ACRG subtypes, we performed a set of exploratory analyses on 167 TCGA samples classified into four distinct subtypes (MSI, EMT, TP53+, and TP53−) [8]. GSEA has shown that EMT-type tumors did indeed exhibit hallmarks of epithelial–mesenchymal transition (False Discovery Rate (FDR) = 0.038) and angiogenesis (FDR = 0.047) as their top enrichment signals. Other subtypes, however, have consistently shown G2M checkpoint and E2F/MYC targets as their top enrichment results (FDR < 0.05) (Supplementary Figure S1). This suggests a more profound difference in the transcriptional rewiring of EMT-type tumors compared to other subtypes. 

Next, we reconstructed PPI networks, highlighting interactions among the differentially expressed genes in each subtype compared to normal samples (adjusted *p*-value ≤ 0.05, absolute LFC ≥ 3). We then identified and compared the top-scoring modules of the different subtypes based on the greedy algorithm of the jActiveModules Cytoscape plug-in. Considerable overlap between the top modules of MSI, TP53+, and TP53− subtypes was observed, yet the top module of the EMT subtype did not share any genes with the other subtypes (Supplementary Figure S2).

Finally, the results of the PCA on the complete expression matrices of TCGA tumors revealed that the samples belonging to the EMT subtype are roughly distinguished in PC1; this is while no tangible difference can be observed between the other three subtypes (Supplementary Figure S3). In accordance with our observations in the TCGA samples, similar results were also observed in the PCA of the ACRG samples (Supplementary Figure S3).

Overall, these results indicated that the samples belonging to the EMT subtype display the most distinct transcriptional profile among all the ACRG subtypes.

### 3.2. WGCNA and Motif Ranking Identify 39 Core mRNA Motifs

**Figure 1:**
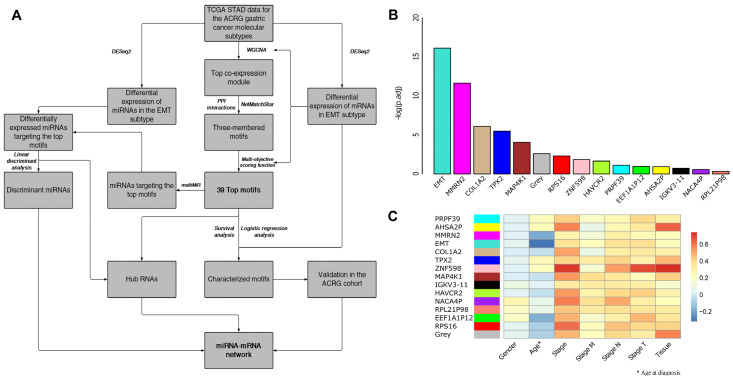
Workflow and co-expression modules. (**A**) Schematic presentation of the overall workflow of this study; (**B**) Bar plot of module significance (defined here as the minus logarithm of the adjusted *p*-values of the differential expression of all the members of a module in the epithelia-mesenchymal transition (EMT) subtype in comparison to the pooled samples of the other subtypes); (**C**) Association of the co-expression modules with clinical parameters. There is a significant negative correlation between the eigengene of the EMT module and the age at diagnosis (R = −0.31; *p*-value = 0.03). It should be noted that since all of the co-expression modules were identified on the same set of samples, the observation that the eigengene of the EMT module is negatively correlated with the age at diagnosis is not biased by possible age imbalances in the data. Tumor staging system: T—size and spread of the primary tumor; N—level of spread to lymph nodes; M—metastasis.

In order to find robust prognostic/diagnostic RNA markers, we sought to take advantage of co-expression module and motif identification approaches to identify core RNA regulators of EMT-type tumors. The workflow implemented for the identification of these RNAs is shown in Figure 1A. Fourteen co-expression modules with varying numbers of genes were identified by applying WGCNA on the expression data of the 47 EMT-type tumors in the TCGA cohort. A list of members of each module is provided in Supplementary Table S3. We used the negative logarithm of each gene’s adjusted *p*-value, after differential expression analysis between EMT-type samples and other subtypes, as the criterion for gene significance. Using this criterion, the module with the most significant average differential expression was designated as the “EMT” module and the members of this module were selected for further investigation (Figure 1B). Since a high level of module membership indicates that the expression level of a gene is an adequate proxy for the general behavior of a module, the label for the rest of the modules was based on the gene with the highest level of module membership in that module. The association of the eigengenes of each module with clinical parameters (gender, age at diagnosis, pathological stage, TNM stages, and the tissue of origin) was also assessed (Figure 1C). There is a significant negative correlation between the eigengene of the EMT module and the age at diagnosis, suggesting the potential role of the members of this module in the earlier onset of the disease.

Triangle motifs (with three nodes and three edges) are the most common type of motifs and are known to largely regulate the higher network structures and serve as the core building blocks of complex biological networks [20,30]. To identify core regulatory elements of the EMT module by taking advantage of the biological relevance of triangle motifs, the PPI network of the members of this module was reconstructed in Cytoscape. A total of 920 triangle motifs were identified. Each one of these motifs was scored based on 13 different weighting scenarios (Supplementary Table S2) using the multi-objective scoring function (see Section 2). Supplementary Table S4 contains all 920 motifs with their corresponding scores in each of the weighting scenarios. The top 10 motifs based on each of the weighting scenarios were selected. After removing the redundant motifs, a total of 39 top motifs remained and were used for further evaluation (Table 2). These motifs represent potent candidates for playing central roles in GC, specifically the EMT subtype. This is due to the fact that the utilized scoring function was designed to designate the best scores to the motifs with the most profound topological significance, diagnostic value, and differential expression in the EMT subtype in comparison to the other subtypes.

### 3.3. Expression of the DMD/ITGA1/CAV1 Motif Is a Strong Predictor of Patient Survival

**Figure 2:**
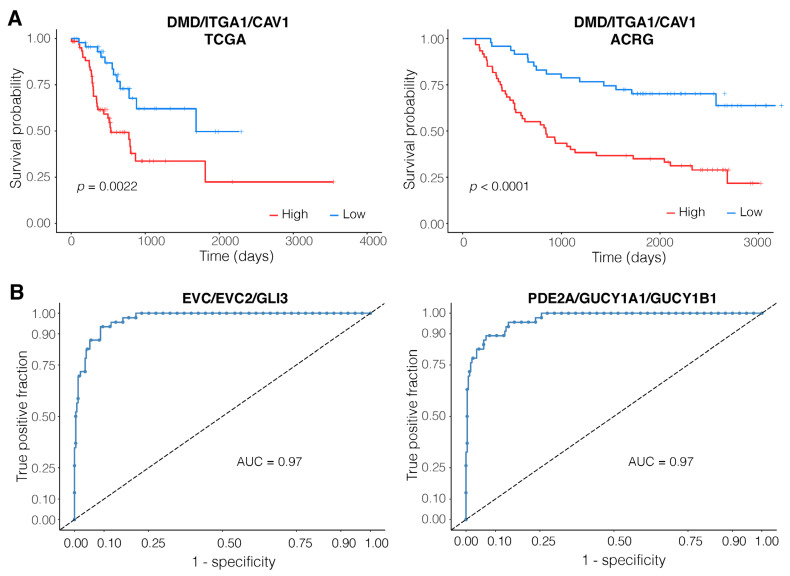
Diagnostic and prognostic capacities of the identified top motifs and members of the miRNA-based diagnostic model. (**A**) DMD/ITGA1/CAV1 was identified as the top motif with consistent relevance to prognosis in both TCGA (left) and ACRG (right) cohorts (hazard ratio > 3 in both cohorts); (**B**) Receiver Operating Characteristic (ROC) curves of the top motifs with diagnostic relevance in the validation set (ACRG cohort). For the complete set of plots for TCGA survival analysis, ACRG survival analysis, and ROC curves, refer to Supplementary Figures S4–S6.

Next, we set out to characterize the 39 top motifs and identify the most potent candidates in terms of their prognostic capability. To this end, we conducted a survival analysis on the motifs based on the expression levels of the members of the motifs. For each member of the motifs, and for each motif considered a single entity, samples were divided into high expression and low expression groups both for the TCGA and ACRG cohorts, Kaplan–Meier curves were constructed (Figure 2A), and multivariate cox regression results (to account for the effects of age and sex) were extracted (Table 2). Considering our stringent criteria (Section 2), the DMD/ITGA1/CAV1 motif was identified as the top motif with consistent relevance to prognosis (HR > 3 in both TCGA and ACRG cohorts).

### 3.4. EVC/EVC2/GLI3 and PDE2A/GUCY1A1/GUCY1B1 Are Robust Diagnostic Motifs

In order to assess the diagnostic capacity of the motifs and identify the most significant motifs with diagnostic relevance, we conducted a logistic regression analysis. Members of the motifs were used as predictors and the subtype of the samples (EMT versus non-EMT) as the response variable. We used the TCGA cohort as the training set and the ACRG cohort as the validation set. Additionally, the independently normalized data from the samples of the Singapore cohort were used to assess the robustness of the models. The top two motifs based on their Area Under the Receiver Operating Characteristic Curve (AUC) in the validation set were EVC/EVC2/GLI3 (AUC = 0.97) and PDE2A/GUCY1A1/GUCY1B1 (AUC = 0.97) (Figure 2B; Table 3). We also assessed the diagnostic capacity of the motifs for distinguishing tumors from normal samples using the data from TCGA-STAD normal and tumor tissues and the GSE184336 dataset as an independent test set. Interestingly, PDE2A/GUCY1A1/GUCY1B1 achieved the highest AUC in the TCGA cohort (AUC = 0.95) and an AUC of 0.85 in the test set of the GSE184336 dataset, reinforcing its diagnostic importance (Table 4).

### 3.5. A Two-Membered miRNA Model Accurately Distinguishes EMT-Type Tumors from Other Gastric Tumors

**Figure 3:**
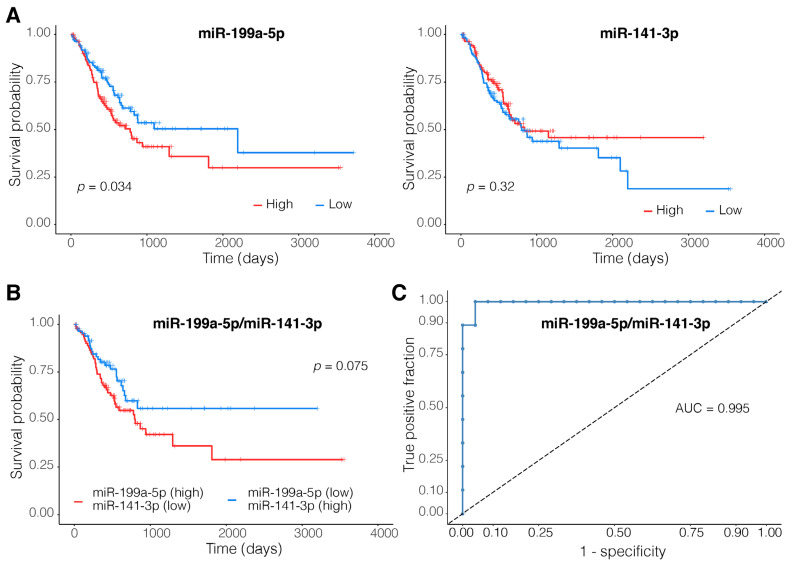
Top two-membered miRNA combination. Kaplan-Meier plots of (**A**) miRNAs model components and (**B**) their combination. Only the expression levels of hsa-miR-199a-5p are significantly associated with patient prognosis. (**C**) The two-membered miRNA-based diagnostic model presents an almost perfect Area Under the Receiver Operating Characteristic Curve (AUC) of 0.995 in the validation set.

The candidate miRNAs regulating the expression of the identified motifs were determined through the identification of differentially expressed miRNAs (EMT vs other subtypes; *n* = 220) that targeted one or more genes among the members of the top 39 motifs (109 miRNAs). The top multi-candidate miRNA combination was identified using LDA with leave-one-out cross-validation. The top two-membered miRNA combination consisting of hsa-miR-199a-5p and hsa-miR-141-3p with an AUC of 0.963 in the training set and an AUC of 0.995 in the test set was identified as the best discriminant multi-candidate miRNA combination (index: (0.597167 × hsa-miR-199a-5p) + (−0.798247 × hsa-miR-141-3p) + 2.02755). The results of the survival analysis for these miRNAs and their combination are demonstrated in Figure 3.

**Figure 4:**
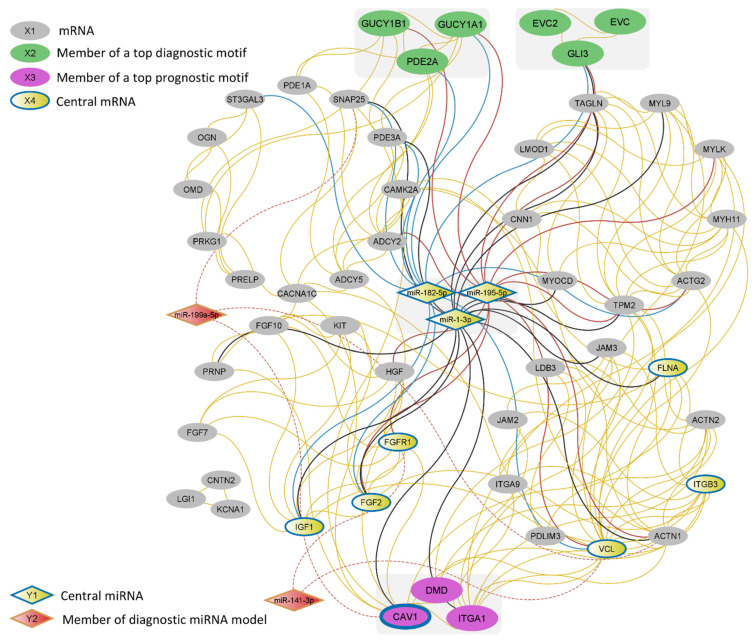
A network of top motifs from the 13 motif ranking scenarios and their miRNA regulators. The top 10 central RNAs based on the Latora closeness [31] are marked by blue margins. Yellow edges represent protein–protein interactions. MiR-182-5p, miR-195-5p, miR-1-3p interactions are represented with blue, red, and black solid lines, respectively. The interactions of the members of the miRNA diagnostic model are represented by dashed lines. To reduce complexity, only the 3 miRNAs that were among the top 10 central RNAs and the two miRNAs from the multi-candidate discriminatory miRNA combination are shown. The complete interaction data of the network consisting of 109 miRNAs, 51 mRNAs, and 435 edges are available in Supplementary Table S7.

Finally, the integrated interaction network of the members of the top 39 motifs and the 109 differentially expressed miRNAs targeting them was visualized (Figure 4).

## 4. Discussion

Among the molecular classifications of gastric tumors by ACRG, tumors of the EMT subtype are associated with significantly worse patient prognosis and likely demand more drastic therapeutic interventions [9]. Coupling this with the vastly unknown nature of the tumors of this subtype, further investigation of the molecular landscape of these tumors and the development of diagnostic and predictive biomarkers are of utmost importance. Here, we have identified a differentially expressed co-expression network in the tumors of the EMT subtype using WGCNA. The negative correlation of this module with the age of the patients at the time of diagnosis (Figure 1C) is in line with the characterization of this subtype by ACRG [8] and indicates the relevance of this module to the EMT subtype. We have further explored this co-expression module in order to extract its central motifs and regulatory miRNAs with relevance to diagnosis and prognosis.

### 4.1. Poor Outcomes for Patients with High Expressions of DMD/ITGA1/CAV1 Motif

Our results are able to characterize the signaling circuits involved in the aggressive phenotypes often observed in the gastric tumors of the EMT subtype (e.g., invasion, chemoresistance, etc.). We have identified the DMD/ITGA1/CAV1 motif as the top motif with consistent relevance to prognosis (HR > 3 in both TCGA and ACRG cohorts). The *ITGA1* gene encodes the α-1 subunit of the integrin superfamily of glycoproteins. These transmembrane receptors are responsible for a variety of cellular functions including cell adhesion, migration, and intracellular signaling in response to the extracellular environment (ECM) [32]. ITGA1 is extensively associated with cancer invasiveness and poor patient prognosis in various tumor types. It promotes EMT, proliferation, and drug resistance in response to dysregulations in the tumor extracellular matrix. This is in part realized through upregulation of the Ras/MEK/ERK (MAPK) pathway [33,34,35,36]. Additionally, a wealth of studies indicate that the EMT-promoting effects of dysregulation in various molecules in GC converge on ITGA1, highlighting its potential as a therapeutic target [37,38].

Upon stimulation, the integrin receptors activate Ras through the recruitment of the Grb2/SOS complex. This is a process in which Caveolin-1 (Cav-1), a protein encoded by another member of the identified motif (*CAV1*), has been shown to play a pivotal role [39]. Cav-1 is best known for its crucial roles as a component of the caveolae—invaginations in the cell membrane involved, among other functions, in cell surface receptor localization and signal transduction [40]. Similar to ITGA1, Cav-1 is strongly associated with poor treatment outcomes, poor prognosis, and EMT [41,42]. Importantly, MAPK is not the only pathway through which Cav-1 has been associated with EMT. It has been shown that Cav-1 stimulates the dephosphorylation of β-Catenin, culminating in the activation of the WNT pathway and upregulation of Met receptor tyrosine kinase. Met (also known as HGFR), through its positive crosstalk with HER2, contributes to tumor aggressiveness, migration, proliferation, and chemoresistance by upregulating MAPK, WNT, and PI3K/AKT pathways [40]. Studies investigating the role of DMD, the last member of the identified motif, are sparse and contradictory [43], warranting a need for further investigation of the role of the DMD in the GC EMT subtype and its functional association with ITGA1 and Cav-1.

### 4.2. The EVC/EVC2/GLI3 Motif Performs Well Both as a Diagnostic and a Prognostic Marker

Our analysis pipeline resulted in the identification of two motifs with superior relevance to the diagnosis of gastric tumors of the EMT subtype. The top identified motif consists of *EVC*, *EVC2*, and *GLI3*; genes coding for essential members of the Hedgehog (Hh) signaling pathway [44]. The Hh pathway is firmly associated with the exhibition of stem-like phenotypes in cancer, cancer cell migration, EMT, and drug resistance in various cancer types including GC [45,46,47]. GLI3 is a transcription factor central to the regulation of the Hh pathway and plays dual roles both as an activator and a repressor of the genes downstream of this pathway [44]. In the absence of the Hh pathway ligands, GLI3 is bound to SUFU, which mediates its proteolytic cleavage, resulting in the abundance of cleaved GLI3 proteins, which act as suppressors of the Hh pathway. In the presence of the Hh ligands, SUFU dissociates from the GLI3 in a process in which both EVC and EVC2 have been shown to be of vital importance [48]. The dissociated full-length GLI3 promotes upregulation of the Hh pathway. The activity of GLI3 is strongly associated with various malignancies. For example, it promotes proliferation and EMT in multiple cancer types [49,50] and plays a role as a cancer driver gene in GC [51]. Importantly, multiple lines of evidence associate the overexpression of GLI3 with poor prognosis in various tumor types [50,52]. In line with these reports, our results indicate considerably worse outcomes for patients with higher expression of the EVC/EVC2/GLI3 motif in both TCGA (HR = 2) and ACRG (HR = 2.7) cohorts, suggesting the possible utility of this motif as a prognostic indicator as well as a diagnostic marker.

### 4.3. PDE2A/GUCY1A1/GUCY1B1—A Strong Diagnostic Marker

The other identified top motif with potential diagnostic capacity for the EMT subtype of GC is comprised of PDE2A (a member of the phosphodiesterase superfamily), GUCY1A1, and GUCY1B1 (also known as GUCY1A3 and GUCY1B3, respectively). These molecules are central regulators of the metabolism of cyclic guanosine monophosphate (cGMP) and cyclic adenosine monophosphate (cAMP), secondary messengers involved in many cellular functions including cell proliferation, differentiation, and apoptosis [53]. Interestingly, in addition to its exceptional performance in discriminating the samples of the EMT subtype from other gastric tumors, this motif presented a capacity for distinguishing gastric tumors from normal samples (AUC = 0.95; highest AUC among the assessed motifs), demonstrating its potential use as a diagnostic marker of GC in general. Notably, the presence of other proteins of the phosphodiesterase superfamily (PDE1A and PDE3A) and adenylate cyclase 5 (ADCY5) in addition to guanylate cyclase (GUCY) proteins among the identified top motifs (Table 3; Figure 4) points to a likely central role of cAMP and cGMP metabolism in the EMT subtype of GC. In line with this, there are a plethora of studies indicating the viability of phosphodiesterase inhibition as a treatment approach for the suppression of proliferation and reduction of the invasion capacity of tumors in various cancers [54]. However, the exact role of these molecules in tumorigenesis and cancer progression is ambiguous, and specifically, the interplay between the cyclase and phosphodiesterase proteins in cancer remains largely unexplored.

### 4.4. MiR-199a-5p and miR-141-3p Dysregulations Are Associated with Tumor Invasiveness

Another important result of this study is the identification of a candidate two-membered miRNA diagnostic biomarker (AUC = 0.995; Figure 3) consisting of hsa-miR-199a-5p (upregulated in the samples of the EMT subtype; LFC = 1.4) and hsa-miR-141-3p (downregulated in the samples of the EMT subtype; LFC = −1.9). In contrast to its downregulation in various tumor types, the expression of hsa-miR-199a is shown to be increased in the case of GC and has been associated with increased tumor invasiveness and metastasis in multiple studies [55,56]. These reports are in accordance with the observations of the current study and support the positive coefficient of this molecule in the identified diagnostic model. The other member of our two-membered diagnostic model, hsa-miR-141-3p, is a member of the miR-200 family of miRNAs, the downregulation of the members of which is tightly associated with increased proliferation, EMT, and invasiveness of gastric tumors among other tumor types [57,58,59]. Altogether, these results highly support the relevance of the identified two-membered miRNA-based diagnostic model in distinguishing gastric tumors of the EMT subtype. Additionally, the expression of both of these miRNAs was associated with patient outcomes in GC in previous studies [55,59]. However, our results only indicate a positive association between the high expression of hsa-miR-199a-5p and poor survival (*p*-value = 0.034). No association between the expression of hsa-miR-141-3p and patient prognosis could be observed (*p*-value = 0.34; Figure 3). 

## 5. Conclusions

A few points regarding the implemented methods for motif identification and their limitations in this study should be noted. Considering the effects of multi-collinearity, the coefficients in the logistic regression modeling of the motifs should be utilized with caution when inferring the behavior of the mRNAs in these motifs since they are all extracted downstream of WGCNA. Nevertheless, this does not affect the precision of the prediction of the disease status by the motifs, and thus the top motifs with diagnostic capacity represent viable candidates. One should also take note that, based on the design of this study, the identified motifs are inclined to be more important in the EMT subtype, but their importance is not necessarily restricted to it; especially due to the inclusion of weighting factors such as the topological significance and previous association with cancer pathways in the motif ranking procedure. Additionally, while the top motifs in terms of prognostic and diagnostic capacity were the main focus of this discussion, all of the other high-scoring motifs in different weighting scenarios (Supplementary Table S4) represent potential candidates for playing significant roles in the pathology of GC and are encouraged to be further explored. Finally, this investigation was carried out entirely in silico, and subsequent wet-lab experiments are necessary for further validation of the results.

Overall, the current study took advantage of the biological relevance of both co-expression modules and network motifs through the combination of their identification methods in an end-to-end analysis workflow. Exploiting the abilities of WGCNA, a multi-objective motif scoring function, and machine learning approaches, we identified combinations of mRNAs and regulatory miRNAs with considerable prognostic and diagnostic capability. These results highlight the central roles of MAPK, Hh, and cGMP/cAMP signaling in the pathology of the EMT subtype of GC and provide an unprecedented picture of rewired signaling circuits that possibly contribute to the phenotypes observed in tumors of this subtype. Additionally, the identified co-expression modules and the large number of characterized motifs provide an opportunity for further exploration of this subtype of gastric tumors through various study designs.

## Figures and Tables

**Table 1:** The distribution of the clinical information within each subtype for the TCGA-STAD, ACRG, and Singapore cohorts.

Cohort	Subtype	Sample Size	Age (Mean ± sd)	Sex		AJCC Pathologic Stage			
				Male	Female	I	II	III	IV
TCGA-STAD	*EMT*	47	61.7 ± 10.06	62%	38%	7%	33%	51%	9%
	*MSI*	37	70.16 ± 10.58	54%	46%	27%	32%	30%	11%
	*TP53+*	42	66.44 ± 11.02	71%	29%	15%	41%	39%	5%
	*TP53*−	41	66.92 ± 9.59	68%	32%	23%	36%	31%	10%
	*NA*	149	66.77 ± 11.02	64%	36%	13%	27%	48%	12%
ACRG	*EMT*	46	55.72 ± 12.44	59%	41%	4%	15%	39%	41%
	*MSI*	68	64.82 ± 9.94	66%	34%	21%	38%	28%	13%
	*TP53+*	78	61.86 ± 11.67	72%	28%	5%	38%	34%	23%
	*TP53*−	105	62.86 ± 10.48	65%	35%	8%	32%	31%	30%
Singapore	*EMT*	83	62.64 ± 13.15	60%	40%	11%	16%	36%	37%
	*MSI*	11	69.33 ± 12.67	55%	45%	36%	9%	36%	18%
	*TP53+*	37	63.15 ± 13.2	73%	27%	19%	16%	38%	27%
	*TP53*−	61	66.58 ± 13.24	69%	31%	18%	15%	39%	28%

**Table 2:** The results of Cox regression analysis for the 39 top motifs.

Node1	Node2	Node3	HR in TCGA	Cox Regression *p*-Value in TCGA	HR in ACRG	Cox Regression *p*-Value in ACRG
ACTN2	LDB3	PDLIM3	1.199	0.51	2.936	0.019
ADCY5	CAV1	CACNA1C	2.396	0.007	4.406	>0.001
CAMK2A	ADCY5	CACNA1C	1.853	0.054	1.234	0.585
CAMK2A	ACTN1	CACNA1C	2.499	0.003	0.958	0.919
CAMK2A	ADCY5	ADCY2	1.716	0.077	0.994	0.989
CNN1	MYH11	ACTG2	1.608	0.042	2.179	>0.001
DMD	ITGA1	CAV1	3.636	>0.001	3.13	>0.001
EVC	EVC2	GLI3	2.035	0.007	2.746	>0.001
FLNA	ITGB3	CAV1	2.997	0.001	2.088	0.019
FLNA	ITGB3	VCL	2.438	0.01	2.299	0.03
GUCY1A1	GUCY1B1	PDE3A	1.716	0.034	1.905	0.006
GUCY1A1	GUCY1B1	PRKG1	1.852	0.012	1.76	0.02
IGF1	FGF7	FGFR1	2.133	0.009	2.47	0.001
IGF1	FGF10	FGFR1	1.926	0.02	2.984	0.002
IGF1	FGF10	HGF	2.223	0.009	1.768	0.054
IGF1	FGF10	KIT	1.622	0.102	1.559	0.104
IGF1	FGF2	FGFR1	1.741	0.051	2.303	0.003
IGF1	FGF2	KIT	1.388	0.273	1.377	0.233
IGF1	FGF2	HGF	1.874	0.033	1.663	0.056
IGF1	FGF7	KIT	1.691	0.088	1.489	0.12
ITGA1	ITGB3	CAV1	4.165	>0.001	2.079	0.009
ITGA9	JAM3	JAM2	2.101	0.004	2.13	0.003
ITGB3	VCL	ACTN1	2.45	0.014	1.669	0.22
KCNA1	LGI1	CNTN2	1.184	0.542	1.113	0.782
LMOD1	MYH11	ACTG2	1.534	0.065	2.105	0.002
LMOD1	CNN1	ACTG2	1.599	0.044	1.92	0.004
LMOD1	CNN1	MYH11	1.43	0.113	1.872	0.006
MYH11	MYL9	ACTG2	2.106	0.005	3.318	>0.001
MYH11	TAGLN	ACTG2	1.741	0.03	2.52	>0.001
MYLK	MYH11	ACTG2	1.552	0.071	2.691	>0.001
MYOCD	CNN1	MYH11	1.487	0.096	2.002	0.003
OGN	OMD	PRELP	2.052	0.005	1.48	0.095
OGN	ST3GAL3	OMD	1.614	0.079	1.725	0.069
OGN	ST3GAL3	PRELP	1.797	0.037	2.089	0.017
PDE1A	GUCY1A1	GUCY1B1	1.981	0.009	1.761	0.018
PDE2A	GUCY1A1	GUCY1B1	2.254	0.003	2.23	0.003
PRNP	CAV1	CACNA1C	2.972	0.003	4.006	>0.001
SNAP25	CAV1	CACNA1C	3.014	0.001	3.29	0.001
TPM2	MYH11	ACTG2	1.648	0.069	2.901	>0.001

HR: Hazard Ratio. Note: The complete results of cox regression analysis for each node and motif are available in Supplementary Table S5.

**Table 3:** The diagnostic capacity of the logistic regression models for distinguishing between the EMT subtype and the other subtypes for the 39 top motifs.

Node1	Node2	Node3	AUC in the Training Set (TCGA)	AUC in the Validation Set (ACRG)	AUC in the Independent Set (Singapore)
EVC	EVC2	GLI3	0.943	0.974	0.92
PDE2A	GUCY1A1	GUCY1B1	0.935	0.972	0.947
IGF1	FGF2	FGFR1	0.932	0.969	0.935
ITGA9	JAM3	JAM2	0.944	0.969	0.935
GUCY1A1	GUCY1B1	PDE3A	0.927	0.967	0.944
IGF1	FGF7	FGFR1	0.938	0.967	0.926
GUCY1A1	GUCY1B1	PRKG1	0.927	0.965	0.944
IGF1	FGF10	FGFR1	0.941	0.965	0.932
SNAP25	CAV1	CACNA1C	0.877	0.961	0.904
PRNP	CAV1	CACNA1C	0.9	0.954	0.914
MYLK	MYH11	ACTG2	0.908	0.952	0.913
PDE1A	GUCY1A1	GUCY1B1	0.936	0.949	0.941
ACTN2	LDB3	PDLIM3	0.927	0.948	0.934
IGF1	FGF2	KIT	0.91	0.944	0.9
IGF1	FGF2	HGF	0.911	0.942	0.907
ADCY5	CAV1	CACNA1C	0.893	0.939	0.892
IGF1	FGF7	KIT	0.923	0.937	0.902
MYH11	MYL9	ACTG2	0.904	0.937	0.916
MYH11	TAGLN	ACTG2	0.915	0.935	0.918
DMD	ITGA1	CAV1	0.883	0.929	0.899
OGN	OMD	PRELP	0.935	0.925	0.914
FLNA	ITGB3	VCL	0.888	0.921	0.92
OGN	ST3GAL3	OMD	0.937	0.92	0.893
IGF1	FGF10	KIT	0.931	0.918	0.904
OGN	ST3GAL3	PRELP	0.938	0.916	0.883
FLNA	ITGB3	CAV1	0.897	0.915	0.925
IGF1	FGF10	HGF	0.928	0.915	0.911
LMOD1	CNN1	ACTG2	0.899	0.912	0.876
CNN1	MYH11	ACTG2	0.889	0.911	0.836
ITGA1	ITGB3	CAV1	0.876	0.906	0.904
TPM2	MYH11	ACTG2	0.864	0.882	0.881
ITGB3	VCL	ACTN1	0.815	0.881	0.868
LMOD1	MYH11	ACTG2	0.897	0.877	0.88
LMOD1	CNN1	MYH11	0.887	0.876	0.886
CAMK2A	ACTN1	CACNA1C	0.888	0.864	0.814
MYOCD	CNN1	MYH11	0.847	0.829	0.844
KCNA1	LGI1	CNTN2	0.914	0.783	0.587
CAMK2A	ADCY5	ADCY2	0.887	0.777	0.732
CAMK2A	ADCY5	CACNA1C	0.895	0.765	0.725

AUC: Area Under the Receiver Operating Characteristic Curve. The complete results of all of the logistic regression models, including their *p*-values, area under the receiver operating characteristic curves, and area under the precision-recall curves are available in Supplementary Table S6.

**Table 4:** The diagnostic capacity of the logistic regression models for distinguishing between the normal and gastric cancer tissues for the 39 top motifs.

Node1	Node2	Node3	AUC in the TCGA	AUC in the GSE184336 (Training)	AUC in the GSE184336 (Validation)
PDE2A	GUCY1A1	GUCY1B1	0.95	0.772	0.854
DMD	ITGA1	CAV1	0.932	0.835	0.822
KCNA1	LGI1	CNTN2	0.929	0.83	0.826
PDE1A	GUCY1A1	GUCY1B1	0.92	0.737	0.803
MYLK	MYH11	ACTG2	0.914	0.821	0.884
ITGA9	JAM3	JAM2	0.912	0.663	0.614
ADCY5	CAV1	CACNA1C	0.904	0.696	0.804
IGF1	FGF7	KIT	0.895	0.888	0.868
IGF1	FGF2	KIT	0.893	0.888	0.868
IGF1	FGF10	KIT	0.889	0.887	0.864
ITGA1	ITGB3	CAV1	0.876	0.699	0.757
CAMK2A	ADCY5	CACNA1C	0.858	0.752	0.807
OGN	OMD	PRELP	0.857	0.629	0.691
ACTN2	LDB3	PDLIM3	0.85	0.74	0.709
LMOD1	CNN1	MYH11	0.85	0.707	0.705
OGN	ST3GAL3	OMD	0.849	0.615	0.7
LMOD1	MYH11	ACTG2	0.844	0.682	0.707
LMOD1	CNN1	ACTG2	0.844	0.689	0.677
IGF1	FGF2	HGF	0.831	0.843	0.83
TPM2	MYH11	ACTG2	0.829	0.704	0.72
FLNA	ITGB3	CAV1	0.828	0.691	0.728
MYH11	TAGLN	ACTG2	0.822	0.787	0.812
IGF1	FGF10	HGF	0.821	0.813	0.817
CNN1	MYH11	ACTG2	0.82	0.702	0.701
MYOCD	CNN1	MYH11	0.82	0.698	0.689
PRNP	CAV1	CACNA1C	0.815	0.634	0.69
MYH11	MYL9	ACTG2	0.814	0.751	0.754
SNAP25	CAV1	CACNA1C	0.813	0.74	0.832
OGN	ST3GAL3	PRELP	0.808	0.58	0.606
GUCY1A1	GUCY1B1	PRKG1	0.807	0.741	0.783
CAMK2A	ADCY5	ADCY2	0.792	0.646	0.566
FLNA	ITGB3	VCL	0.768	0.687	0.735
IGF1	FGF2	FGFR1	0.762	0.828	0.776
IGF1	FGF10	FGFR1	0.752	0.773	0.736
CAMK2A	ACTN1	CACNA1C	0.745	0.802	0.811
EVC	EVC2	GLI3	0.734	0.658	0.567
GUCY1A1	GUCY1B1	PDE3A	0.665	0.718	0.748
IGF1	FGF7	FGFR1	0.655	0.791	0.777
ITGB3	VCL	ACTN1	0.613	0.712	0.703

AUC: Area Under the Receiver Operating Characteristic Curve. The complete results of all of the logistic regression models, including their *p*-values, area under the receiver operating characteristic curves, and area under the precision-recall curves are available in Supplementary Table S6.

## Data Availability

All of the datasets used in this study are accessible from the GDC data portal (https://portal.gdc.cancer.gov/ accessed on 21 August 2021) and the Gene Expression Omnibus database (under the accessions GSE62254, GSE15459, and GSE184336).

## Supplementary Materials

The following supporting information can be downloaded at: https://www.mdpi.com/article/10.3390/genes14030750/s1, Supplementary Figure S1: Results of GSEA for all of the gastric cancer subtypes based on the ACRG classification; Supplementary Figure S2: The Venn diagram of the overlap of the members of the top protein–protein interaction modules of different ACRG gastric cancer subtypes; Supplementary Figure S3: Results of PCA for all of the gastric cancer subtypes based on the ACRG classification in both TCGA and ACRG cohorts; Supplementary Figure S4: Kaplan–Meier survival curves of TCGA cohort for all of the top-scoring three-membered motifs as well as each member of the motif in isolation; Supplementary Figure S5: Kaplan–Meier survival curves of the ACRG cohort for all of the top-scoring three-membered motifs as well as each member of the motifs in isolation; Supplementary Figure S6: The receiver operating characteristic (ROC) curves of the diagnostic performance of the models trained on TCGA cohort data in the validation cohort; Supplementary Table S1: Clinical information of TCGA, ACRG, and Singapore cohorts; Supplementary Table S2: 13 weighting scenarios used for motif scoring; Supplementary Table S3: List of the members of all the 14 co-expression modules identified by WGCNA; Supplementary Table S4: All of the 920 identified motifs with their scores in each of the 13 weighting scenarios; Supplementary Table S5: The results of Cox regression analysis for the 39 top motifs in both TCGA and ACRG cohorts; Supplementary Table S6: The performance of the logistic regression models for the 39 top motifs; Supplementary Table S7: Complete miRNA-mRNA network interactions.

## Abbreviations

ACRG, Asian Cancer Research Group; AUC, Area Under the Curve; cAMP, cyclic adenosine monophosphate; cGMP, cyclic guanosine monophosphate; EMT, Epithelial-Mesenchymal Transition; FDR, False Discovery Rate; GSEA, Gene Set Enrichment Analysis; HR, Hazard Ratio; MSI, MicroSatellite Instability; PCA, Principal Component Analysis; PPI, Protein-Protein Interaction; RMA, Robust Multichip Average; ROC, Receiver Operating characteristic Curve, STAD, STomach ADenocarcinoma; TCGA, The Cancer Genome Atlas; VST, Variance Stabilizing Transformation; WGCNA, Weighted Gene Co-expression Network Analysis.

## References

[1] Ho S.W.T., Tan P. (2019). Dissection of Gastric Cancer Heterogeneity for Precision Oncology. Cancer Sci..

[2] Liu Y., Wu J., Huang W., Weng S., Wang B., Chen Y., Wang H. (2020). Development and Validation of a Hypoxia-Immune-Based Microenvironment Gene Signature for Risk Stratification in Gastric Cancer. J. Transl. Med..

[3] Sung H., Ferlay J., Siegel R.L., Laversanne M., Soerjomataram I., Jemal A., Bray F. (2021). Global Cancer Statistics 2020: GLOBOCAN Estimates of Incidence and Mortality Worldwide for 36 Cancers in 185 Countries. CA Cancer J. Clin..

[4] Sanjeevaiah A., Cheedella N., Hester C., Porembka M.R. (2018). Gastric Cancer: Recent Molecular Classification Advances, Racial Disparity, and Management Implications. J. Oncol. Pract..

[5] Laurén P. (1965). The Two Histological Main Types of Gastric Carcinoma: Diffuse and so-called Intestinal-Type Carcinoma. Acta Pathol. Microbiol. Scand..

[6] Bass A.J., Thorsson V., Shmulevich I., Reynolds S.M., Miller M., Bernard B. (2014). Comprehensive Molecular Characterization of Gastric Adenocarcinoma. Nature.

[7] Serra O., Galán M., Ginesta M.M., Calvo M., Sala N., Salazar R. (2019). Comparison and Applicability of Molecular Classifications for Gastric Cancer. Cancer Treat. Rev..

[8] Cristescu R., Lee J., Nebozhyn M., Kim K.-M., Ting J.C., Wong S.S., Liu J., Yue Y.G., Wang J., Yu K. (2015). Molecular Analysis of Gastric Cancer Identifies Subtypes Associated with Distinct Clinical Outcomes. Nat. Med..

[9] Lee J., Cristescu R., Kim K.-M., Kim K., Kim S.T., Park S.H., Kang W.K. (2017). Development of Mesenchymal Subtype Gene Signature for Clinical Application in Gastric Cancer. Oncotarget.

[10] Ooki A., Yamaguchi K. (2022). The Dawn of Precision Medicine in Diffuse-Type Gastric Cancer. Ther. Adv. Med. Oncol..

[11] Zhang Z., Hernandez K., Savage J., Li S., Miller D., Agrawal S., Ortuno F., Staudt L.M., Heath A., Grossman R.L. (2021). Uniform Genomic Data Analysis in the NCI Genomic Data Commons. Nat. Commun..

[12] Love M.I., Huber W., Anders S. (2014). Moderated Estimation of Fold Change and Dispersion for RNA-Seq Data with DESeq2. Genome Biol..

[13] Mootha V.K., Lindgren C.M., Eriksson K.-F., Subramanian A., Sihag S., Lehar J., Puigserver P., Carlsson E., Ridderstråle M., Laurila E. (2003). PGC-1α-Responsive Genes Involved in Oxidative Phosphorylation Are Coordinately Downregulated in Human Diabetes. Nat. Genet..

[14] Ideker T., Ozier O., Schwikowski B., Siegel A.F. (2002). Discovering Regulatory and Signalling Circuits in Molecular Interaction Networks. Bioinformatics.

[15] Tang J., Kong D., Cui Q., Wang K., Zhang D., Gong Y., Wu G. (2018). Prognostic Genes of Breast Cancer Identified by Gene Co-Expression Network Analysis. Front. Oncol..

[16] Langfelder P., Horvath S. (2008). WGCNA: An R Package for Weighted Correlation Network Analysis. BMC Bioinform..

[17] Karimi M.R., Karimi A.H., Abolmaali S., Sadeghi M., Schmitz U. (2022). Prospects and Challenges of Cancer Systems Medicine: From Genes to Disease Networks. Brief. Bioinform..

[18] Szklarczyk D., Gable A.L., Lyon D., Junge A., Wyder S., Huerta-Cepas J., Simonovic M., Doncheva N.T., Morris J.H., Bork P. (2019). STRING V11: Protein–Protein Association Networks with Increased Coverage, Supporting Functional Discovery in Genome-Wide Experimental Datasets. Nucleic Acids Res..

[19] Rinnone F., Micale G., Bonnici V., Bader G.D., Shasha D., Ferro A., Pulvirenti A., Giugno R. (2015). NetMatchStar: An Enhanced Cytoscape Network Querying App. F1000Research.

[20] Alon U. (2007). Network Motifs: Theory and Experimental Approaches. Nat. Rev. Genet..

[21] Khan F.M., Marquardt S., Gupta S.K., Knoll S., Schmitz U., Spitschak A., Engelmann D., Vera J., Wolkenhauer O., Pützer B.M. (2017). Unraveling a Tumor Type-Specific Regulatory Core Underlying E2F1-Mediated Epithelial-Mesenchymal Transition to Predict Receptor Protein Signatures. Nat. Commun..

[22] Sadeghi M., Ordway B., Rafiei I., Borad P., Fang B., Koomen J.L., Zhang C., Yoder S., Johnson J., Damaghi M. (2020). Integrative Analysis of Breast Cancer Cells Reveals an Epithelial-Mesenchymal Transition Role in Adaptation to Acidic Microenvironment. Front. Oncol..

[23] Anaya J. (2016). OncoLnc: Linking TCGA Survival Data to MRNAs, MiRNAs, and LncRNAs. PeerJ Comput. Sci..

[24] Thompson J.A., Tan J., Greene C.S. (2016). Cross-Platform Normalization of Microarray and RNA-Seq Data for Machine Learning Applications. PeerJ.

[25] Yokoi A., Matsuzaki J., Yamamoto Y., Yoneoka Y., Takahashi K., Shimizu H., Uehara T., Ishikawa M., Ikeda S., Sonoda T. (2018). Integrated Extracellular MicroRNA Profiling for Ovarian Cancer Screening. Nat. Commun..

[26] Ru Y., Kechris K.J., Tabakoff B., Hoffman P., Radcliffe R.A., Bowler R., Mahaffey S., Rossi S., Calin G.A., Bemis L. (2014). The MultiMiR R Package and Database: Integration of MicroRNA–Target Interactions along with Their Disease and Drug Associations. Nucleic Acids Res..

[27] Csardi G., Nepusz T. (2006). The Igraph Software Package for Complex Network Research. InterJournal Complex Syst..

[28] Jalili M., Salehzadeh-Yazdi A., Asgari Y., Arab S.S., Yaghmaie M., Ghavamzadeh A., Alimoghaddam K. (2015). CentiServer: A Comprehensive Resource, Web-Based Application and R Package for Centrality Analysis. PLoS ONE.

[29] Ashtiani M., Salehzadeh-Yazdi A., Razaghi-Moghadam Z., Hennig H., Wolkenhauer O., Mirzaie M., Jafari M. (2018). A Systematic Survey of Centrality Measures for Protein-Protein Interaction Networks. BMC Syst. Biol..

[30] Yeger-Lotem E., Sattath S., Kashtan N., Itzkovitz S., Milo R., Pinter R.Y., Alon U., Margalit H. (2004). Network Motifs in Integrated Cellular Networks of Transcription–Regulation and Protein–Protein Interaction. Proc. Natl. Acad. Sci. USA.

[31] Latora V., Marchiori M. (2001). Efficient Behavior of Small-World Networks. Phys. Rev. Lett..

[32] Takada Y., Ye X., Simon S. (2007). The Integrins. Genome Biol..

[33] Gharibi A., La Kim S., Molnar J., Brambilla D., Adamian Y., Hoover M., Hong J., Lin J., Wolfenden L., Kelber J.A. (2017). ITGA1 Is a Pre-Malignant Biomarker That Promotes Therapy Resistance and Metastatic Potential in Pancreatic Cancer. Sci. Rep..

[34] Park E.J., Myint P.K., Ito A., Appiah M.G., Darkwah S., Kawamoto E., Shimaoka M. (2020). Integrin-Ligand Interactions in Inflammation, Cancer, and Metabolic Disease: Insights Into the Multifaceted Roles of an Emerging Ligand Irisin. Front. Cell Dev. Biol..

[35] Braicu C., Buse M., Busuioc C., Drula R., Gulei D., Raduly L., Rusu A., Irimie A., Atanasov A.G., Slaby O. (2019). A Comprehensive Review on MAPK: A Promising Therapeutic Target in Cancer. Cancers.

[36] Hu T., Zhou R., Zhao Y., Wu G. (2016). Integrin A6/Akt/Erk Signaling Is Essential for Human Breast Cancer Resistance to Radiotherapy. Sci. Rep..

[37] Zang D., Zhang C., Li C., Fan Y., Li Z., Hou K., Che X., Liu Y., Qu X. (2020). LPPR4 Promotes Peritoneal Metastasis via Sp1/Integrin α/FAK Signaling in Gastric Cancer. Am. J. Cancer Res..

[38] Yan H., Zheng C., Li Z., Bao B., Yang B., Hou K., Qu X., Xiao J., Che X., Liu Y. (2019). NPTX1 Promotes Metastasis via Integrin/FAK Signaling in Gastric Cancer. Cancer Manag. Res..

[39] Wary K.K., Mariotti A., Zurzolo C., Giancotti F.G. (1998). A Requirement for Caveolin-1 and Associated Kinase Fyn in Integrin Signaling and Anchorage-Dependent Cell Growth. Cell.

[40] Wang X., Lu B., Dai C., Fu Y., Hao K., Zhao B., Chen Z., Fu L. (2020). Caveolin-1 Promotes Chemoresistance of Gastric Cancer Cells to Cisplatin by Activating WNT/β-Catenin Pathway. Front. Oncol..

[41] Nam K.H., Lee B.L., Park J.H., Kim J., Han N., Lee H.E., Kim M.A., Lee H.S., Kim W.H. (2013). Caveolin 1 Expression Correlates with Poor Prognosis and Focal Adhesion Kinase Expression in Gastric Cancer. Pathobiology.

[42] Bailey K.M., Liu J. (2008). Caveolin-1 Up-Regulation during Epithelial to Mesenchymal Transition Is Mediated by Focal Adhesion Kinase. J. Biol. Chem..

[43] Jones L., Naidoo M., Machado L.R., Anthony K. (2021). The Duchenne Muscular Dystrophy Gene and Cancer. Cell. Oncol..

[44] Matissek S.J., Elsawa S.F. (2020). GLI3: A Mediator of Genetic Diseases, Development and Cancer. Cell Commun. Signal..

[45] Xu Y., Song S., Wang Z., Ajani J.A. (2019). The Role of Hedgehog Signaling in Gastric Cancer: Molecular Mechanisms, Clinical Potential, and Perspective. Cell Commun. Signal..

[46] Wang F., Ma L., Zhang Z., Liu X., Gao H., Zhuang Y., Yang P., Kornmann M., Tian X., Yang Y. (2016). Hedgehog Signaling Regulates Epithelial-Mesenchymal Transition in Pancreatic Cancer Stem-Like Cells. J. Cancer.

[47] Fattahi S., Nikbakhsh N., Ranaei M., Sabour D., Akhavan-Niaki H. (2021). Association of Sonic Hedgehog Signaling Pathway Genes IHH, BOC, RAB23a and MIR195-5p, MIR509-3-5p, MIR6738-3p with Gastric Cancer Stage. Sci. Rep..

[48] Caparrós-Martín J.A., Valencia M., Reytor E., Pacheco M., Fernandez M., Perez-Aytes A., Gean E., Lapunzina P., Peters H., Goodship J.A. (2013). The Ciliary Evc/Evc2 Complex Interacts with Smo and Controls Hedgehog Pathway Activity in Chondrocytes by Regulating Sufu/Gli3 Dissociation and Gli3 Trafficking in Primary Cilia. Hum. Mol. Genet..

[49] Rodrigues M.F., Miguita L., De Andrade N., Heguedusch D., Rodini C., Moyses R., Toporcov T., Gama R., Tajara E., Nunes F. (2018). GLI3 Knockdown Decreases Stemness, Cell Proliferation and Invasion in Oral Squamous Cell Carcinoma. Int. J. Oncol..

[50] Li J., Qiu M., An Y., Huang J., Gong C. (2018). MiR-7-5p Acts as a Tumor Suppressor in Bladder Cancer by Regulating the Hedgehog Pathway Factor Gli3. Biochem. Biophys. Res. Commun..

[51] Wang K., Yuen S.T., Xu J., Lee S.P., Yan H.H.N., Shi S.T., Siu H.C., Deng S., Chu K.M., Law S. (2014). Whole-Genome Sequencing and Comprehensive Molecular Profiling Identify New Driver Mutations in Gastric Cancer. Nat. Genet..

[52] Shen M., Zhang Z., Wang P. (2021). GLI3 Promotes Invasion and Predicts Poor Prognosis in Colorectal Cancer. BioMed Res. Int..

[53] Maurice D.H., Ke H., Ahmad F., Wang Y., Chung J., Manganiello V.C. (2014). Advances in Targeting Cyclic Nucleotide Phosphodiesterases. Nat. Rev. Drug Discov..

[54] Peng T., Gong J., Jin Y., Zhou Y., Tong R., Wei X., Bai L., Shi J. (2018). Inhibitors of Phosphodiesterase as Cancer Therapeutics. Eur. J. Med. Chem..

[55] Song G., Zeng H., Li J., Xiao L., He Y., Tang Y., Li Y. (2010). MiR-199a Regulates the Tumor Suppressor Mitogen-Activated Protein Kinase Kinase Kinase 11 in Gastric Cancer. Biol. Pharm. Bull..

[56] Zhang Y., Fan K.-J., Sun Q., Chen A.-Z., Shen W.-L., Zhao Z.-H., Zheng X.-F., Yang X. (2012). Functional Screening for MiRNAs Targeting Smad4 Identified MiR-199a as a Negative Regulator of TGF-β Signalling Pathway. Nucleic Acids Res..

[57] Yu L., Cao C., Li X., Zhang M., Gu Q., Gao H., Balic J.J., Xu D., Zhang L., Ying L. (2022). Complete Loss of MiR-200 Family Induces EMT Associated Cellular Senescence in Gastric Cancer. Oncogene.

[58] Liang Z., Li X., Liu S., Li C., Wang X., Xing J. (2019). MiR-141–3p Inhibits Cell Proliferation, Migration and Invasion by Targeting TRAF5 in Colorectal Cancer. Biochem. Biophys. Res. Commun..

[59] Huang M., Wu L., Qin Y., Li Z., Luo S., Qin H., Yang Y., Chen J. (2016). Anti-Proliferative Role and Prognostic Implication of MiR-141 in Gastric Cancer. Am. J. Transl. Res..

