# Supplementary material for: Network-Based and Machine-Learning Approaches Identify Diagnostic and Prognostic Models for EMT-Type Gastric Tumors
Source: Genes (Basel). 2023 Mar 19;14(3):750. doi: 10.3390/genes14030750 (PMC10048224; doi:10.3390/genes14030750)
Supplement: Supplementary file 1 [file Supplementary Figure S3.pdf › Supplementary Figure S3.pdf]

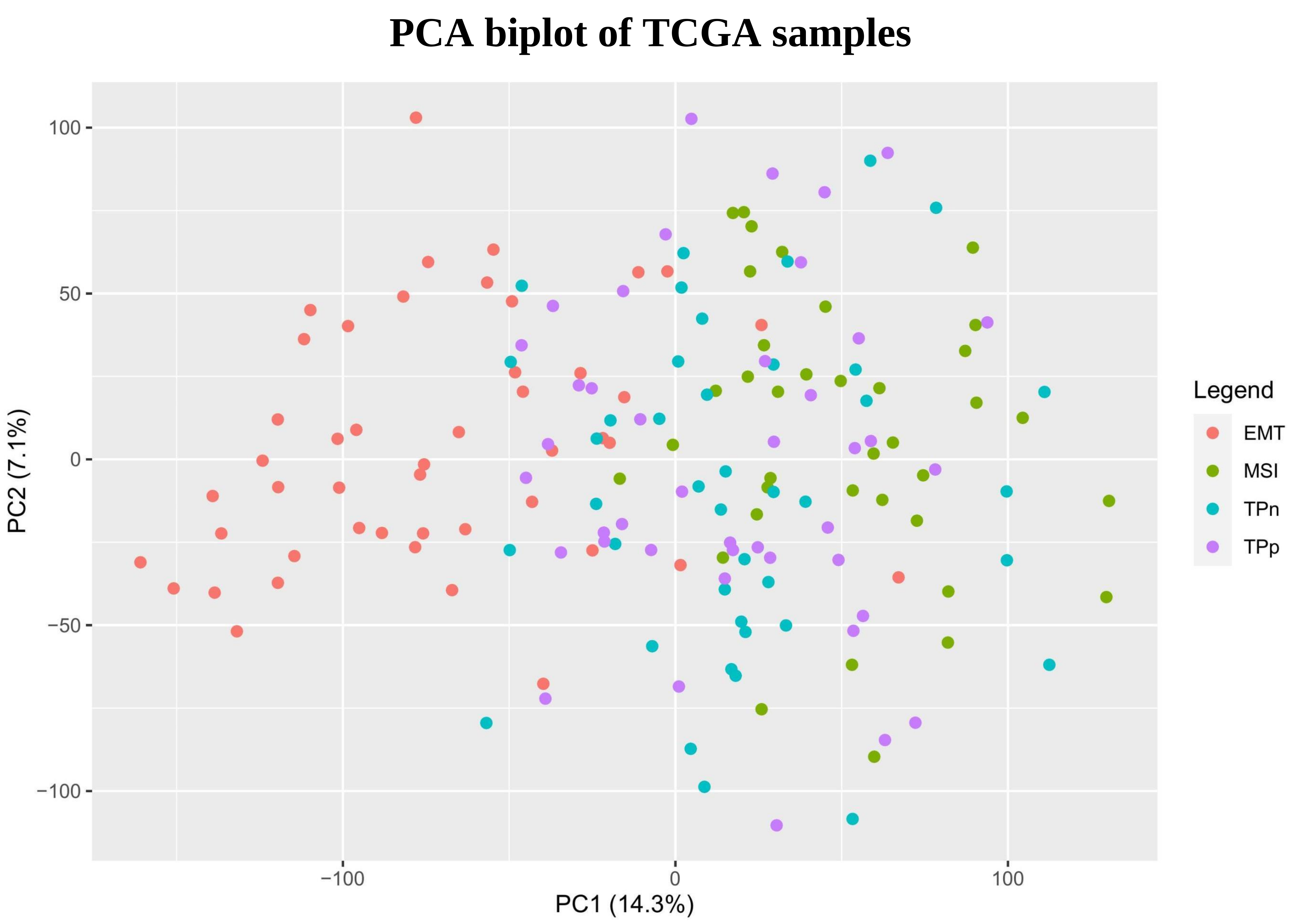

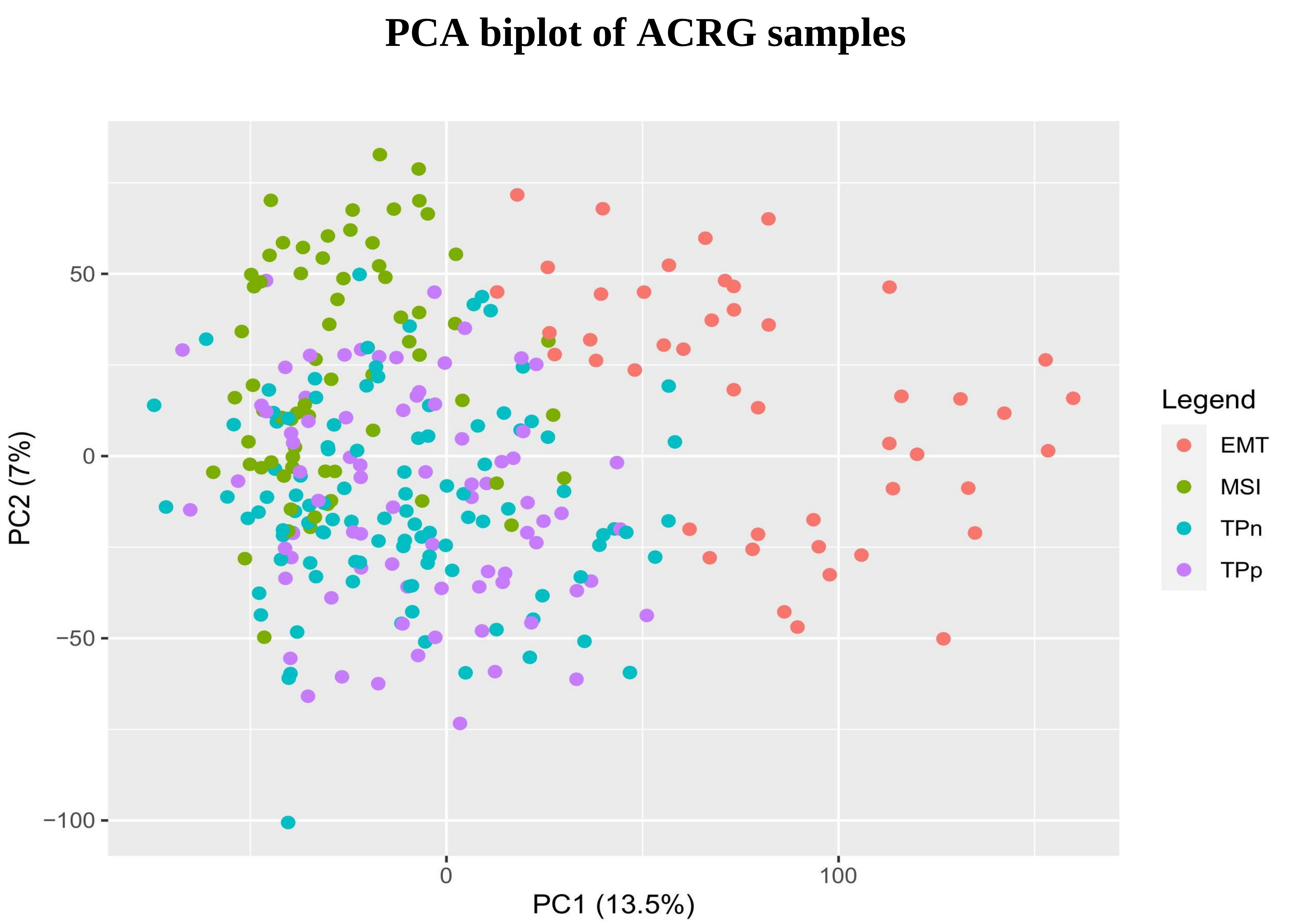

Supplementary Figure S3 Results of PCA for all of the gastric cancer subtypes based on the ACRG classification in both TCGA and ACRG cohorts. These results reinforce that the overall transcriptional profile of the EMT-type tumors presents a more distinct behavior compared to other subtypes as the samples of the EMT subtype can be distinguished on the PC1 axis of the PCA plots
