# Supplementary material for: Network-Based and Machine-Learning Approaches Identify Diagnostic and Prognostic Models for EMT-Type Gastric Tumors
Source: Genes (Basel). 2023 Mar 19;14(3):750. doi: 10.3390/genes14030750 (PMC10048224; doi:10.3390/genes14030750)
Supplement: Supplementary file 1 [file Supplementary Figure S1.pdf › Supplementary Figure S1.pdf]

EMT subtype

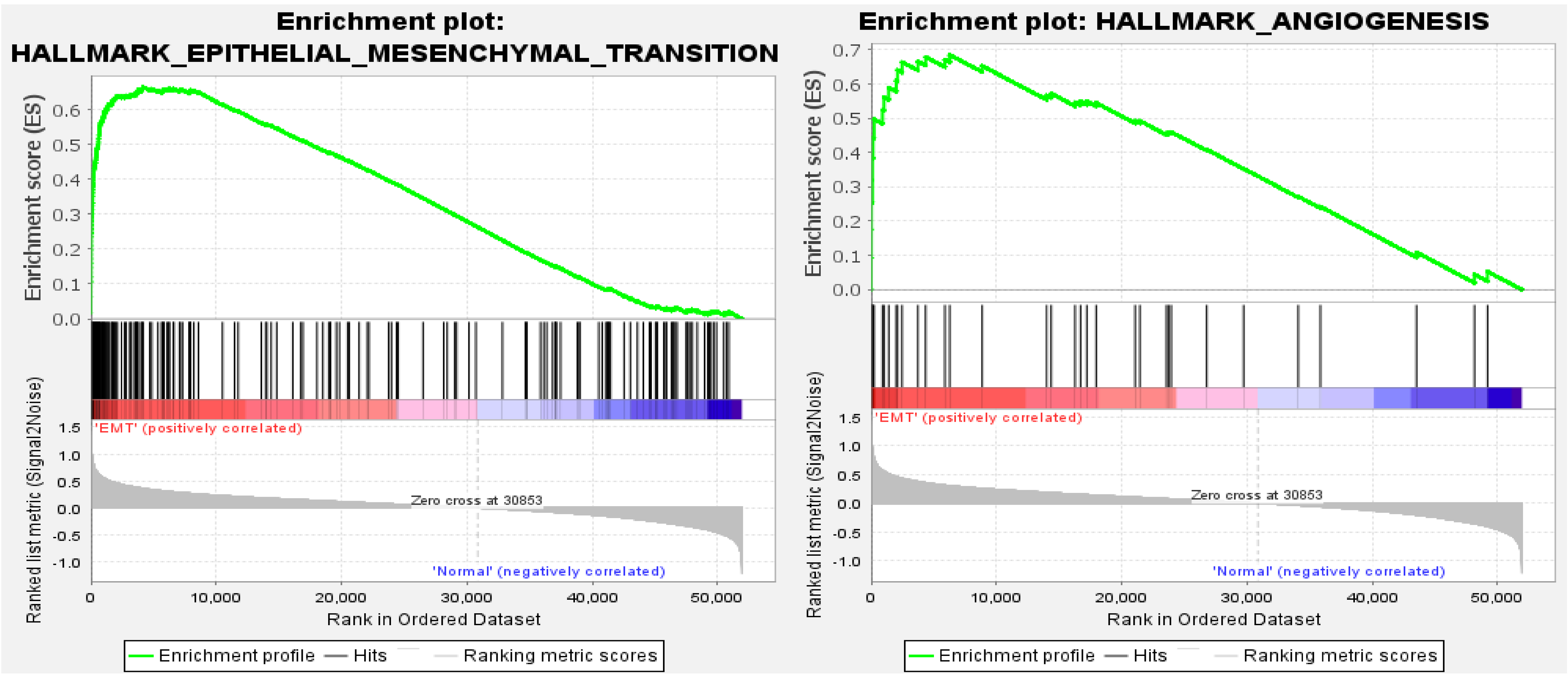

MSI subtype

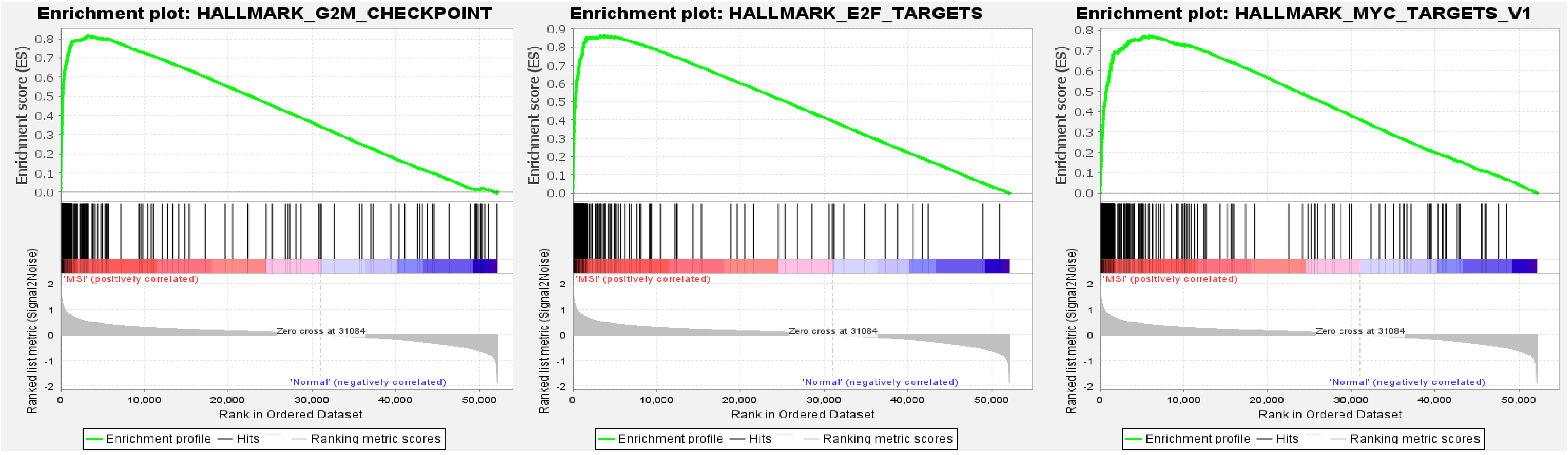

TP53+ subtype

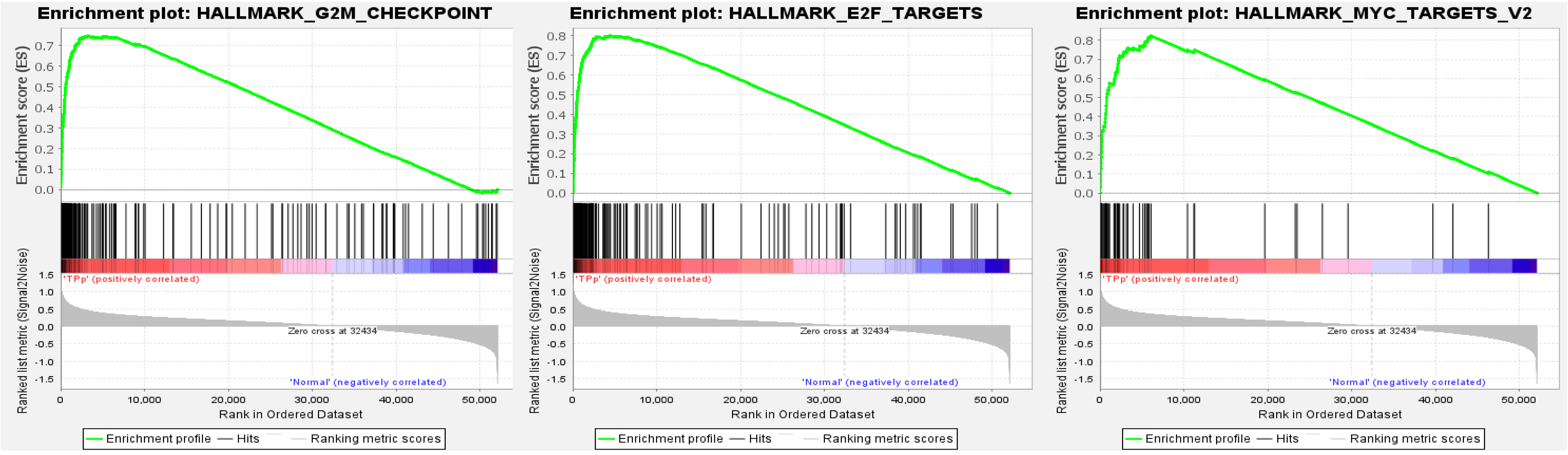

TP53- subtype

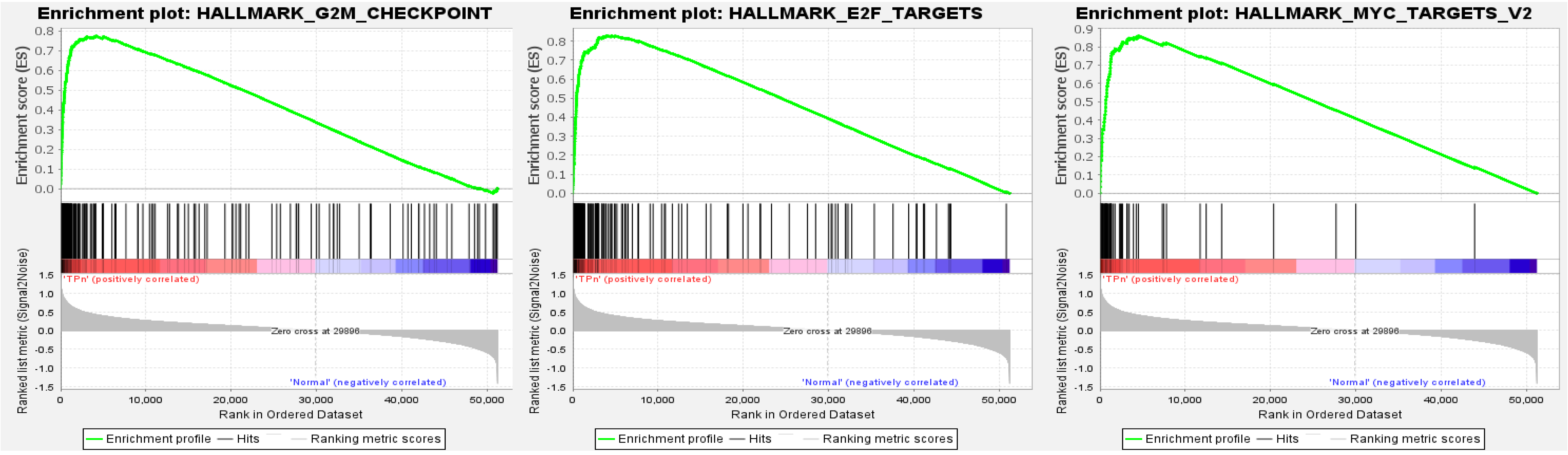

Supplementary Figure S1 Results of GSEA for all of the gastric cancer subtypes based on the ACRG classification. It can be observed that, except for the EMT subtype, the enrichment results for all of the other subtypes are similar, pointing to a more profound difference in the transcriptional landscape of the EMT-type tumors. Additionally, the dominant signals of epithelial-mesenchymal transition and angiogenesis in the EMT samples confirm the relevance of the samples classified in this subtype to the EMT phenotype
