# Supplementary material for: Network-Based and Machine-Learning Approaches Identify Diagnostic and Prognostic Models for EMT-Type Gastric Tumors
Source: Genes (Basel). 2023 Mar 19;14(3):750. doi: 10.3390/genes14030750 (PMC10048224; doi:10.3390/genes14030750)
Supplement: Supplementary file 1 [file genes-14-00750-s001.zip › Supplementary Figure S4-6.pdf]

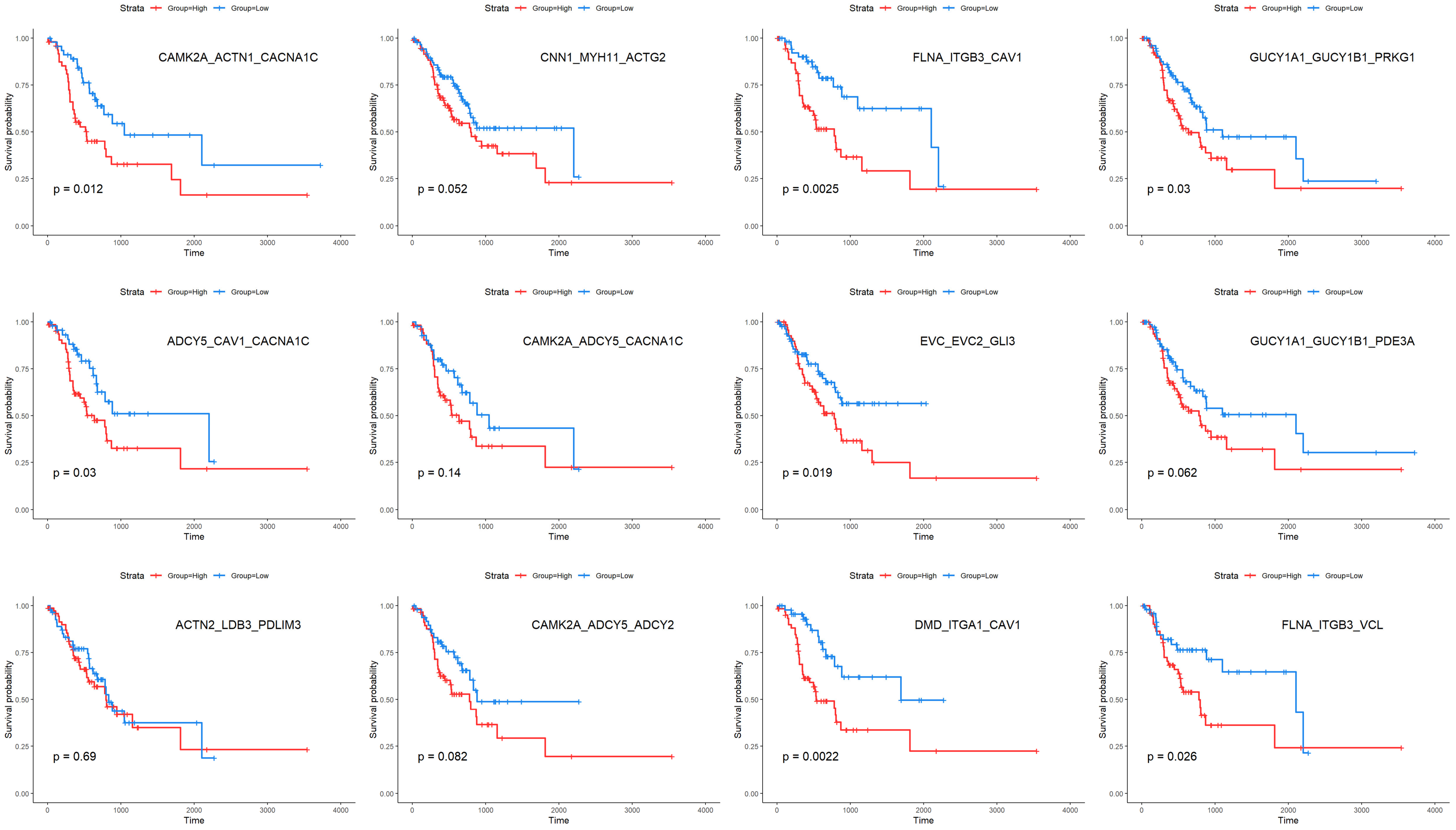
Strata
Group=High
Group=Low
Survival probability
Time
CAMK2A_ACTN1_CACNA1C
p = 0.012
CNN1_MYH11_ACTG2
p = 0.052
FLNA_ITGB3_CAV1
p = 0.0025
GUCY1A1_GUCY1B1_PRKG1
p = 0.03
ADCY5_CAV1_CACNA1C
p = 0.03
CAMK2A_ADCY5_CACNA1C
p = 0.14
EVC_EVC2_GLI3
p = 0.019
GUCY1A1_GUCY1B1_PDE3A
p = 0.062
ACTN2_LDB3_PDLIM3
p = 0.69
CAMK2A_ADCY5_ADCY2
p = 0.082
DMD_ITGA1_CAV1
p = 0.0022
FLNA_ITGB3_VCL
p = 0.026

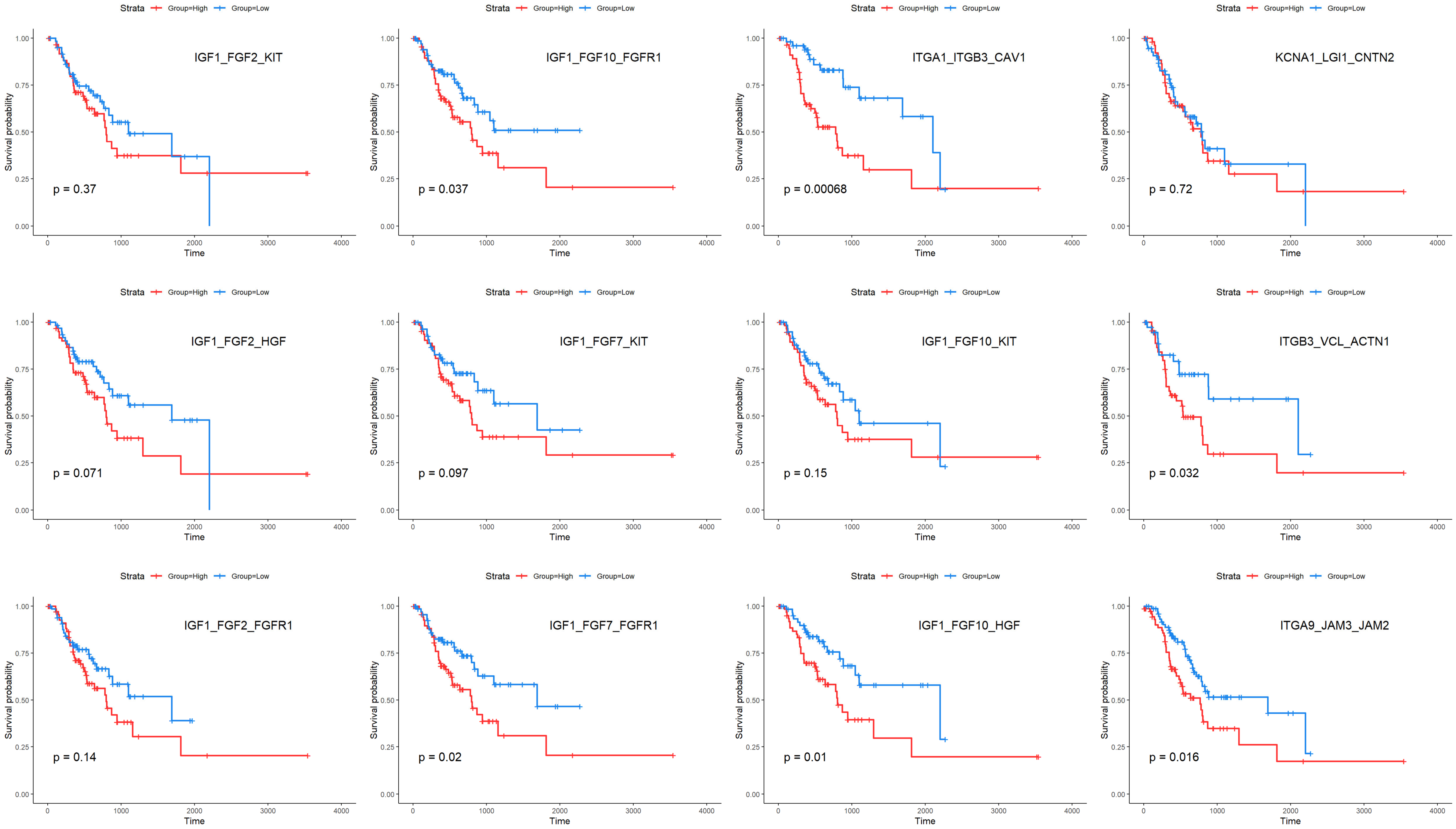
Strata
Group=High
Group=Low
Survival probability
Time
IGF1_FGF2_KIT
p = 0.37
IGF1_FGF10_FGFR1
p = 0.037
ITGA1_ITGB3_CAV1
p = 0.00068
KCNA1_LGI1_CNTN2
p = 0.72
IGF1_FGF2_HGF
p = 0.071
IGF1_FGF7_KIT
p = 0.097
IGF1_FGF10_KIT
p = 0.15
ITGB3_VCL_ACTN1
p = 0.032
IGF1_FGF2_FGFR1
p = 0.14
IGF1_FGF7_FGFR1
p = 0.02
IGF1_FGF10_HGF
p = 0.01
ITGA9_JAM3_JAM2
p = 0.016

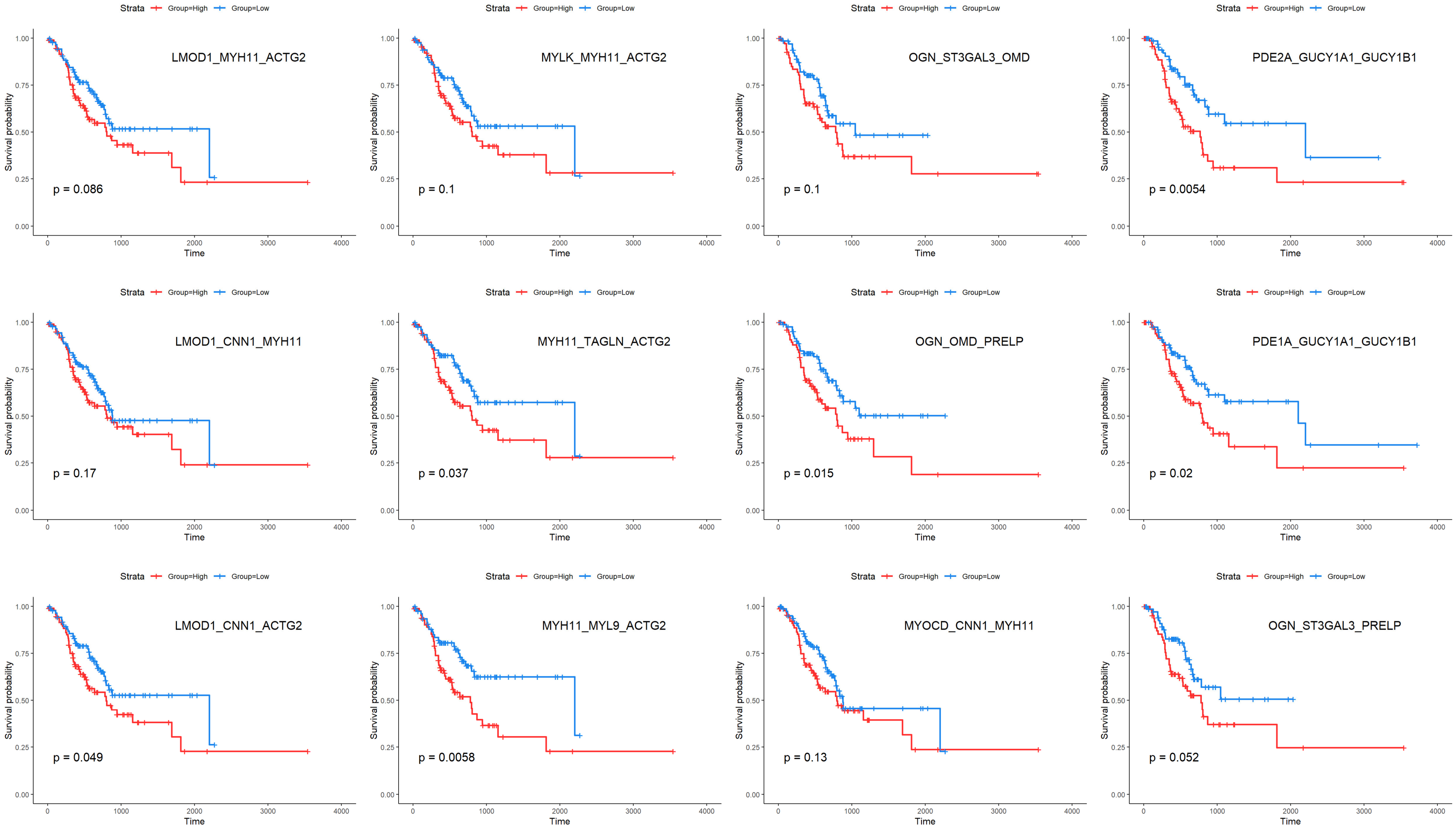

Strata
Group=High
Group=Low
Survival probability
Time
1.00
0.75
0.50
0.25
0.00
0
1000
2000
3000
4000
LMOD1_MYH11_ACTG2
p = 0.086
MYLK_MYH11_ACTG2
p = 0.1
OGN_ST3GAL3_OMD
p = 0.1
PDE2A_GUCY1A1_GUCY1B1
p = 0.0054
LMOD1_CNN1_MYH11
p = 0.17
MYH11_TAGLN_ACTG2
p = 0.037
OGN_OMD_PRELP
p = 0.015
PDE1A_GUCY1A1_GUCY1B1
p = 0.02
LMOD1_CNN1_ACTG2
p = 0.049
MYH11_MYL9_ACTG2
p = 0.0058
MYOCD_CNN1_MYH11
p = 0.13
OGN_ST3GAL3_PRELP
p = 0.052

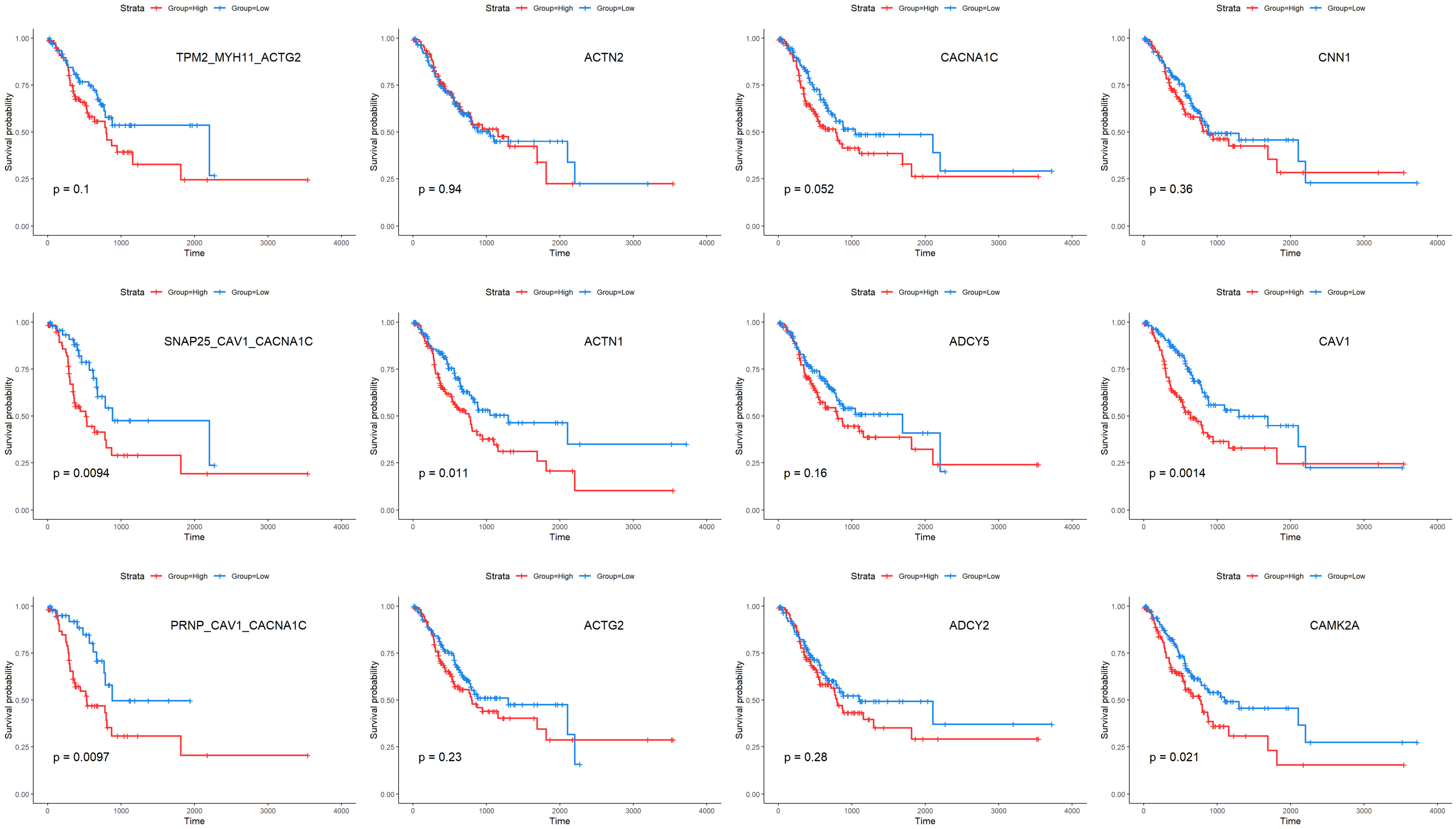

Strata
Group=High
Group=Low
TPM2_MYH11_ACTG2
p = 0.1
ACTN2
p = 0.94
CACNA1C
p = 0.052
CNN1
p = 0.36
SNAP25_CAV1_CACNA1C
p = 0.0094
ACTN1
p = 0.011
ADCY5
p = 0.16
CAV1
p = 0.0014
PRNP_CAV1_CACNA1C
p = 0.0097
ACTG2
p = 0.23
ADCY2
p = 0.28
CAMK2A
p = 0.021
Survival probability
1.00
0.75
0.50
0.25
0.00
Time
0
1000
2000
3000
4000

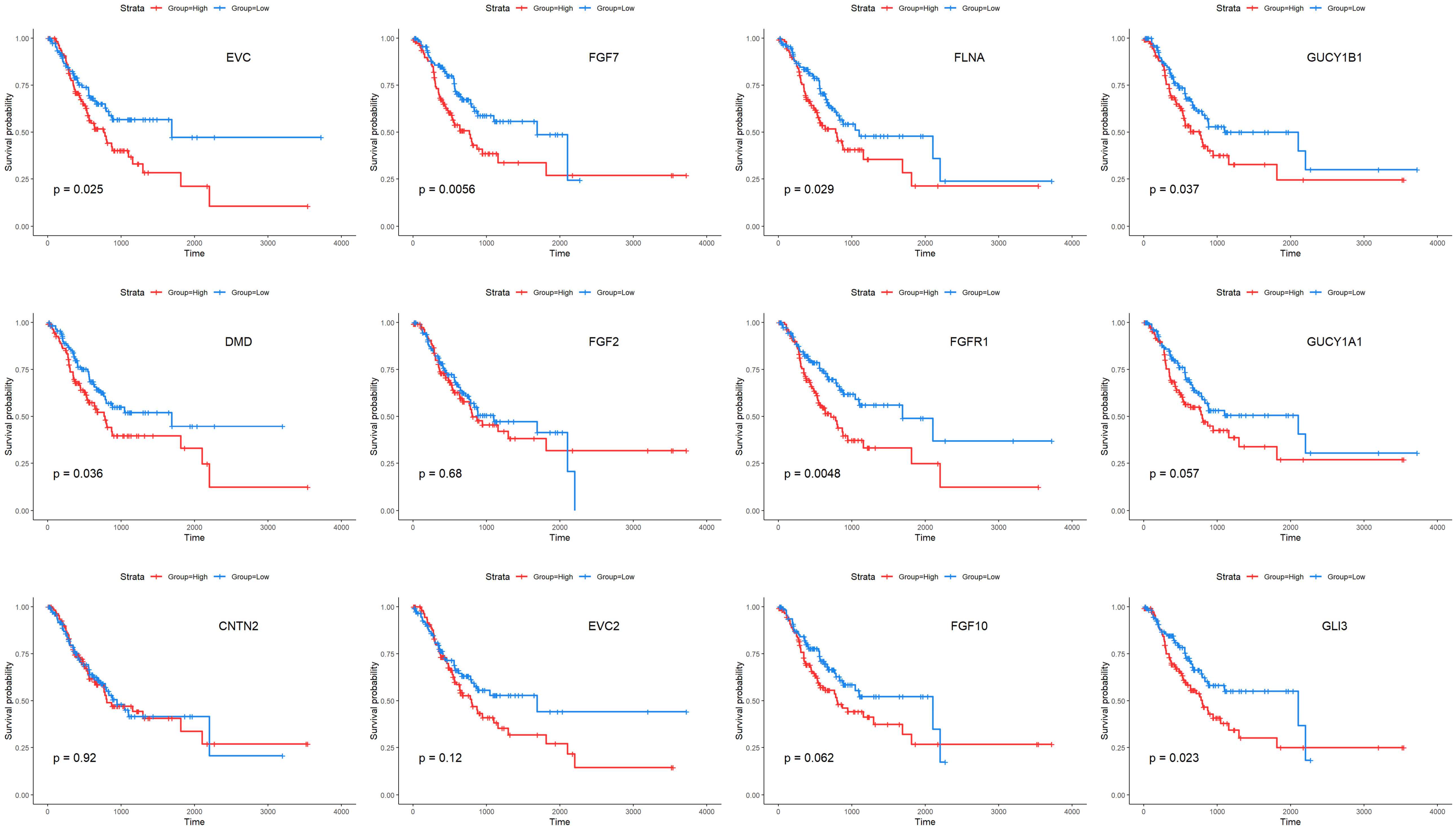

Strata Group=High Group=Low
EVC
p = 0.025
FGF7
p = 0.0056
FLNA
p = 0.029
GUCY1B1
p = 0.037
DMD
p = 0.036
FGF2
p = 0.68
FGFR1
p = 0.0048
GUCY1A1
p = 0.057
CNTN2
p = 0.92
EVC2
p = 0.12
FGF10
p = 0.062
GLI3
p = 0.023
Survival probability
1.00
0.75
0.50
0.25
0.00
Time
0
1000
2000
3000
4000

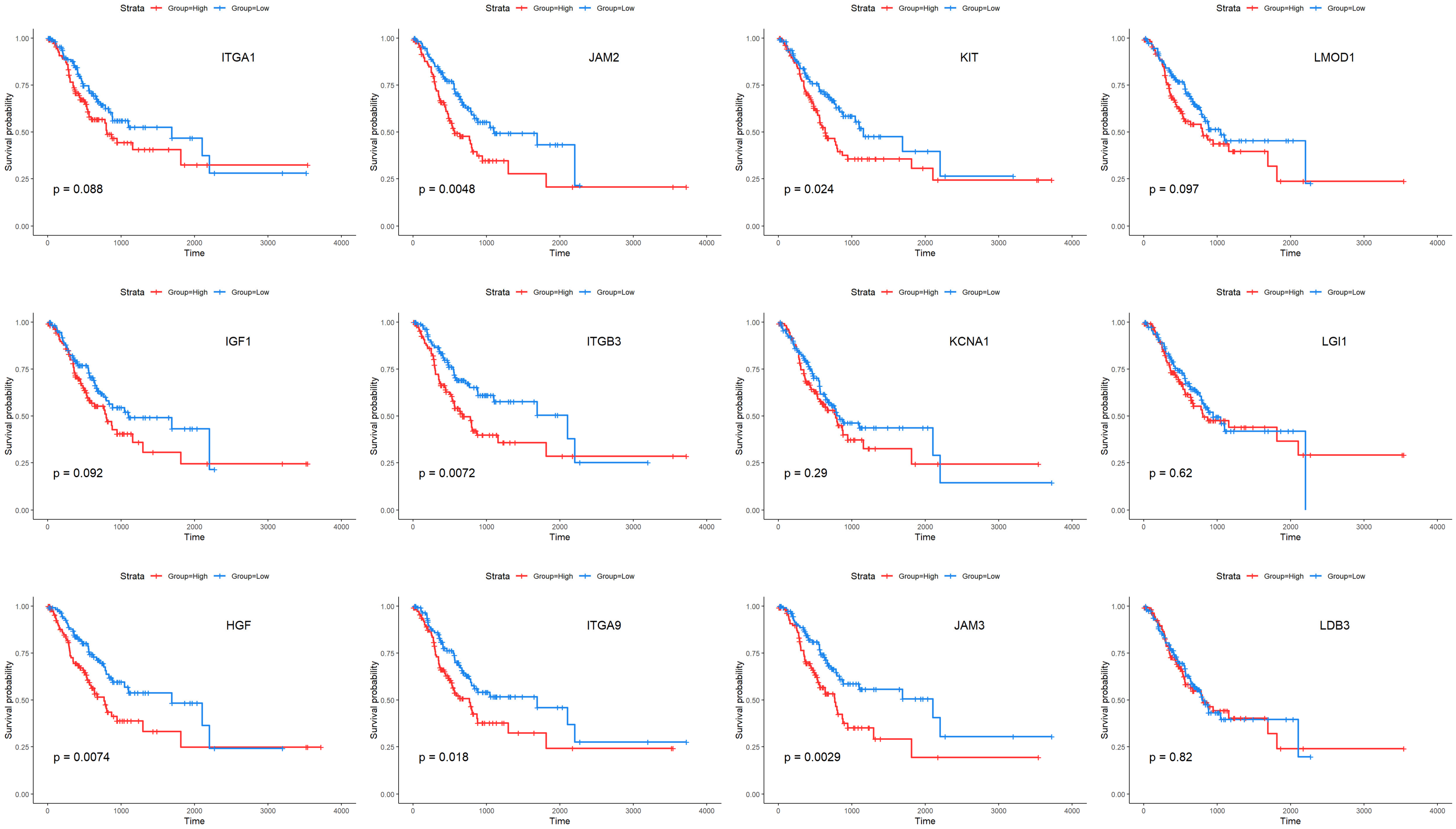
Strata
Group=High
Group=Low
Survival probability
Time
ITGA1
p = 0.088
JAM2
p = 0.0048
KIT
p = 0.024
LMOD1
p = 0.097
IGF1
p = 0.092
ITGB3
p = 0.0072
KCNA1
p = 0.29
LGI1
p = 0.62
HGF
p = 0.0074
ITGA9
p = 0.018
JAM3
p = 0.0029
LDB3
p = 0.82

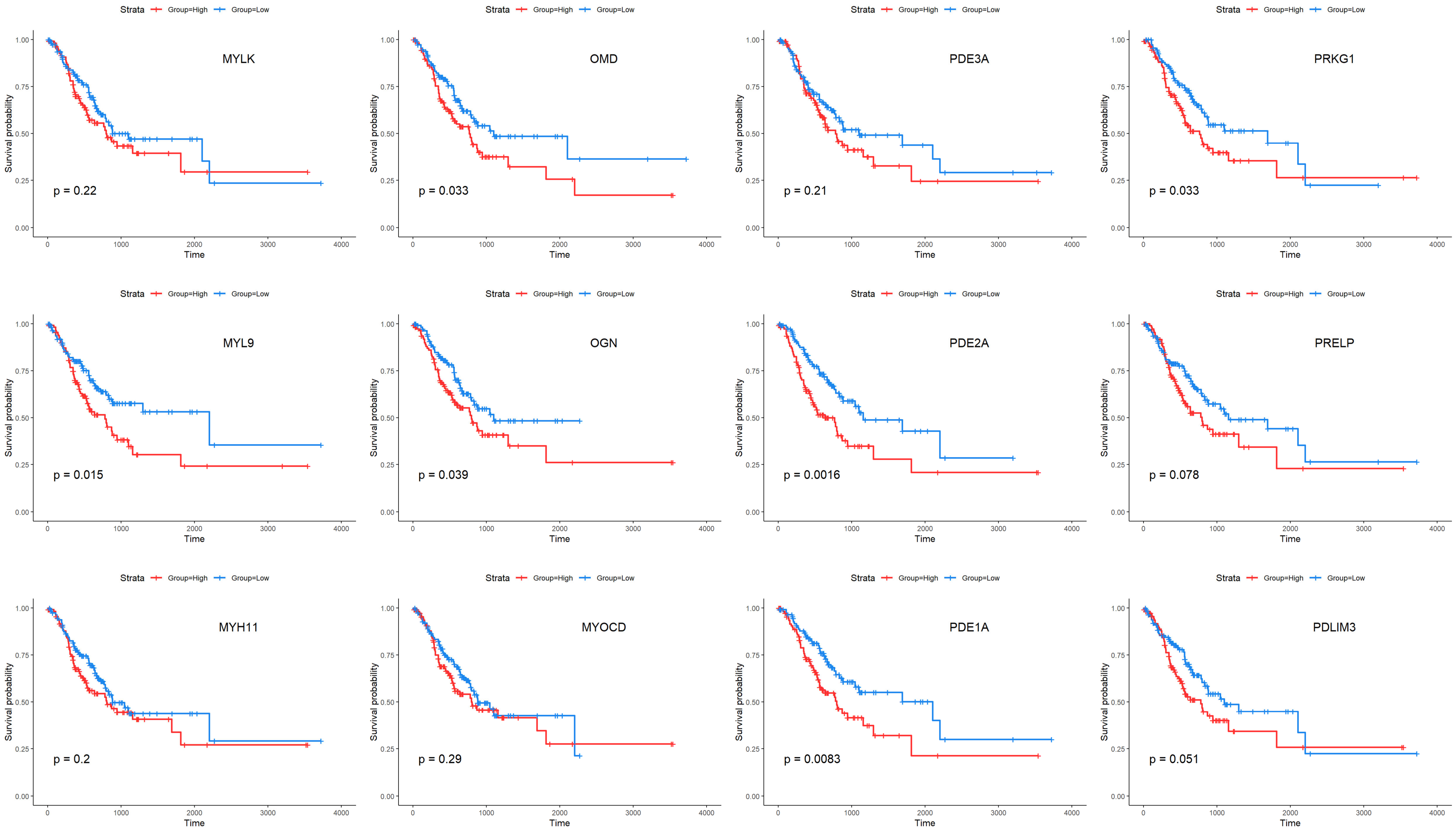
Strata Group=High Group=Low
Survival probability
Time
MYLK
p = 0.22
OMD
p = 0.033
PDE3A
p = 0.21
PRKG1
p = 0.033
MYL9
p = 0.015
OGN
p = 0.039
PDE2A
p = 0.0016
PRELP
p = 0.078
MYH11
p = 0.2
MYOCD
p = 0.29
PDE1A
p = 0.0083
PDLIM3
p = 0.051

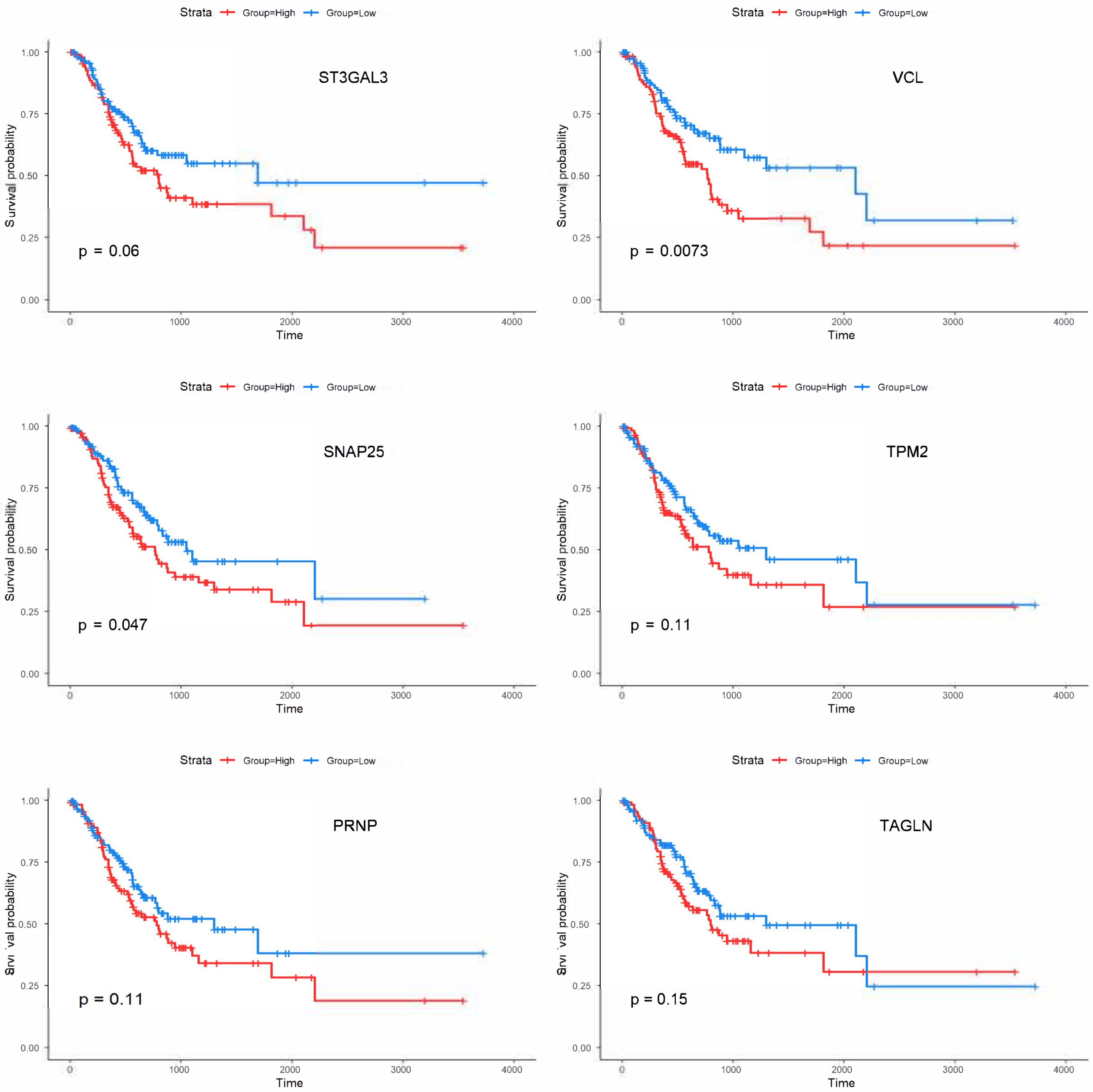

Supplementary Figure S4 Kaplan-Meier survival curves of TCGA cohort for all of the top-scoring three-membered motifs as well as each member of the motifs in isolation. For motif members, the 'High' and 'Low' groups represent the top and bottom 40% of the samples in terms of the expression of the respective genes. For the whole motifs, the intersection of the samples among the top 40% of samples and the bottom 40% of samples based on the expression of each of the three members of the respective motifs was used to form 'High' and 'low' groups

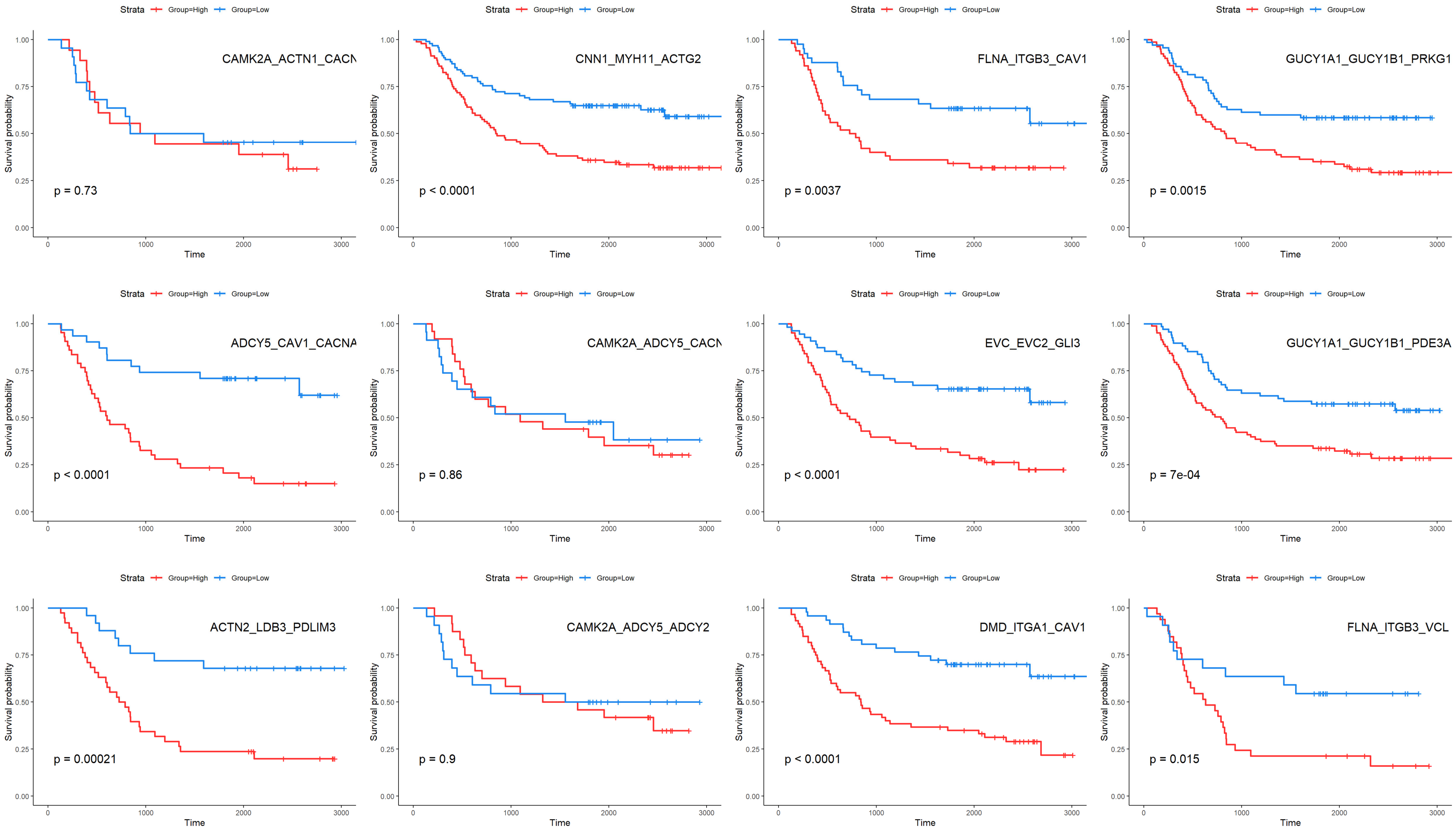
Strata Group=High Group=Low
CAMK2A_ACTN1_CACN
p = 0.73
CNN1_MYH11_ACTG2
p < 0.0001
FLNA_ITGB3_CAV1
p = 0.0037
GUCY1A1_GUCY1B1_PRKG1
p = 0.0015
ADCY5_CAV1_CACNA
p < 0.0001
CAMK2A_ADCY5_CACN
p = 0.86
EVC_EVC2_GLI3
p < 0.0001
GUCY1A1_GUCY1B1_PDE3A
p = 7e-04
ACTN2_LDB3_PDLIM3
p = 0.00021
CAMK2A_ADCY5_ADCY2
p = 0.9
DMD_ITGA1_CAV1
p < 0.0001
FLNA_ITGB3_VCL
p = 0.015
Survival probability
Time
0.00 0.25 0.50 0.75 1.00
0 1000 2000 3000

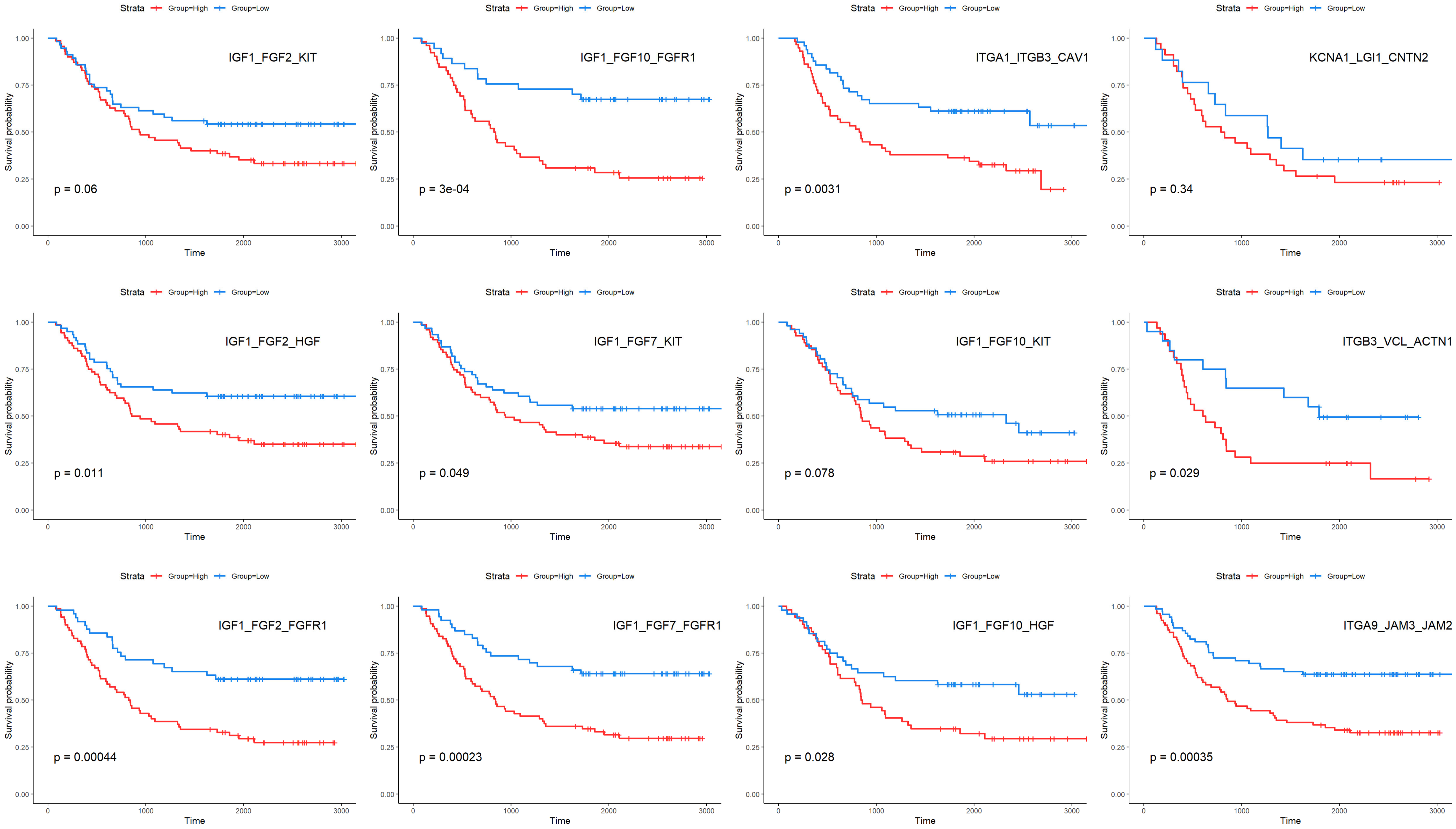
IGF1_FGF2_KIT
p = 0.06
IGF1_FGF10_FGFR1
p = 3e-04
ITGA1_ITGB3_CAV1
p = 0.0031
KCNA1_LGI1_CNTN2
p = 0.34
IGF1_FGF2_HGF
p = 0.011
IGF1_FGF7_KIT
p = 0.049
IGF1_FGF10_KIT
p = 0.078
ITGB3_VCL_ACTN1
p = 0.029
IGF1_FGF2_FGFR1
p = 0.00044
IGF1_FGF7_FGFR1
p = 0.00023
IGF1_FGF10_HGF
p = 0.028
ITGA9_JAM3_JAM2
p = 0.00035
Strata
Group=High
Group=Low
Survival probability
Time

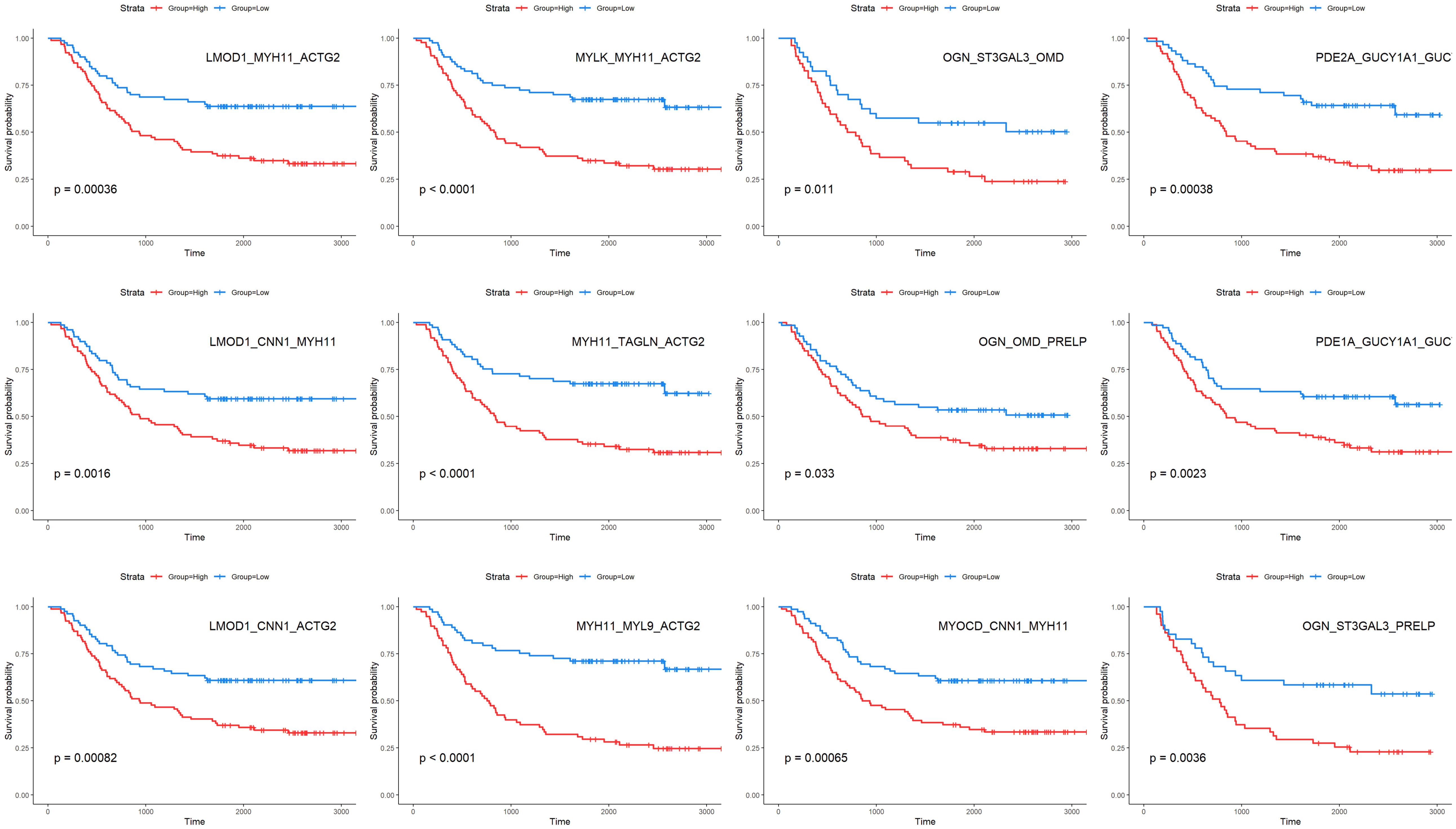
Strata
Group=High
Group=Low
LMOD1_MYH11_ACTG2
p = 0.00036
MYLK_MYH11_ACTG2
p < 0.0001
OGN_ST3GAL3_OMD
p = 0.011
PDE2A_GUCY1A1_GUC
p = 0.00038
LMOD1_CNN1_MYH11
p = 0.0016
MYH11_TAGLN_ACTG2
p < 0.0001
OGN_OMD_PRELP
p = 0.033
PDE1A_GUCY1A1_GUC
p = 0.0023
LMOD1_CNN1_ACTG2
p = 0.00082
MYH11_MYL9_ACTG2
p < 0.0001
MYOCD_CNN1_MYH11
p = 0.00065
OGN_ST3GAL3_PRELP
p = 0.0036
Survival probability
1.00
0.75
0.50
0.25
0.00
Time
0
1000
2000
3000

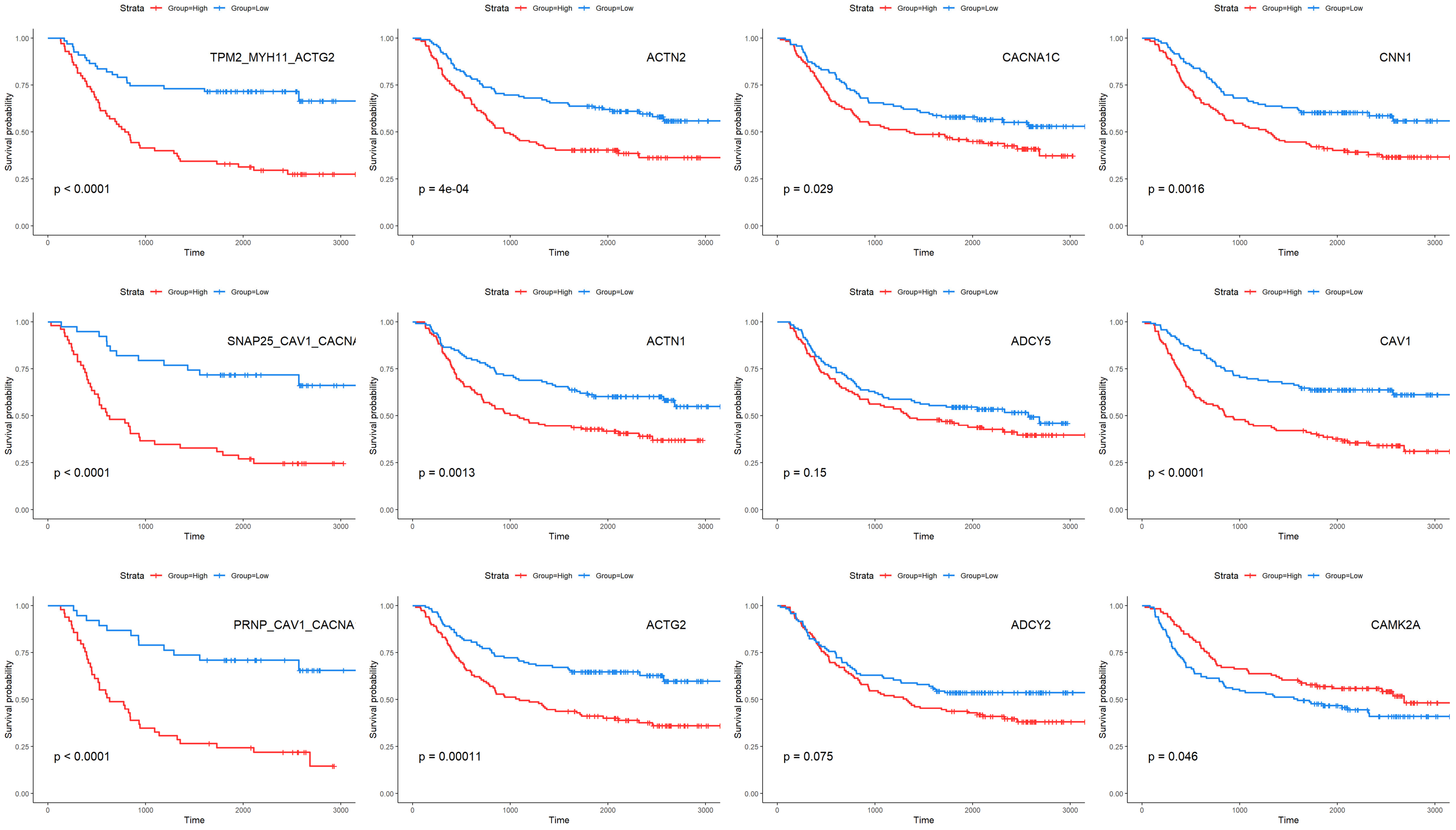
Strata
Group=High
Group=Low
Survival probability
Time
TPM2_MYH11_ACTG2
p < 0.0001
ACTN2
p = 4e-04
CACNA1C
p = 0.029
CNN1
p = 0.0016
SNAP25_CAV1_CACN/
p < 0.0001
ACTN1
p = 0.0013
ADCY5
p = 0.15
CAV1
p < 0.0001
PRNP_CAV1_CACNA
p < 0.0001
ACTG2
p = 0.00011
ADCY2
p = 0.075
CAMK2A
p = 0.046
1.00
0.75
0.50
0.25
0.00
0
1000
2000
3000

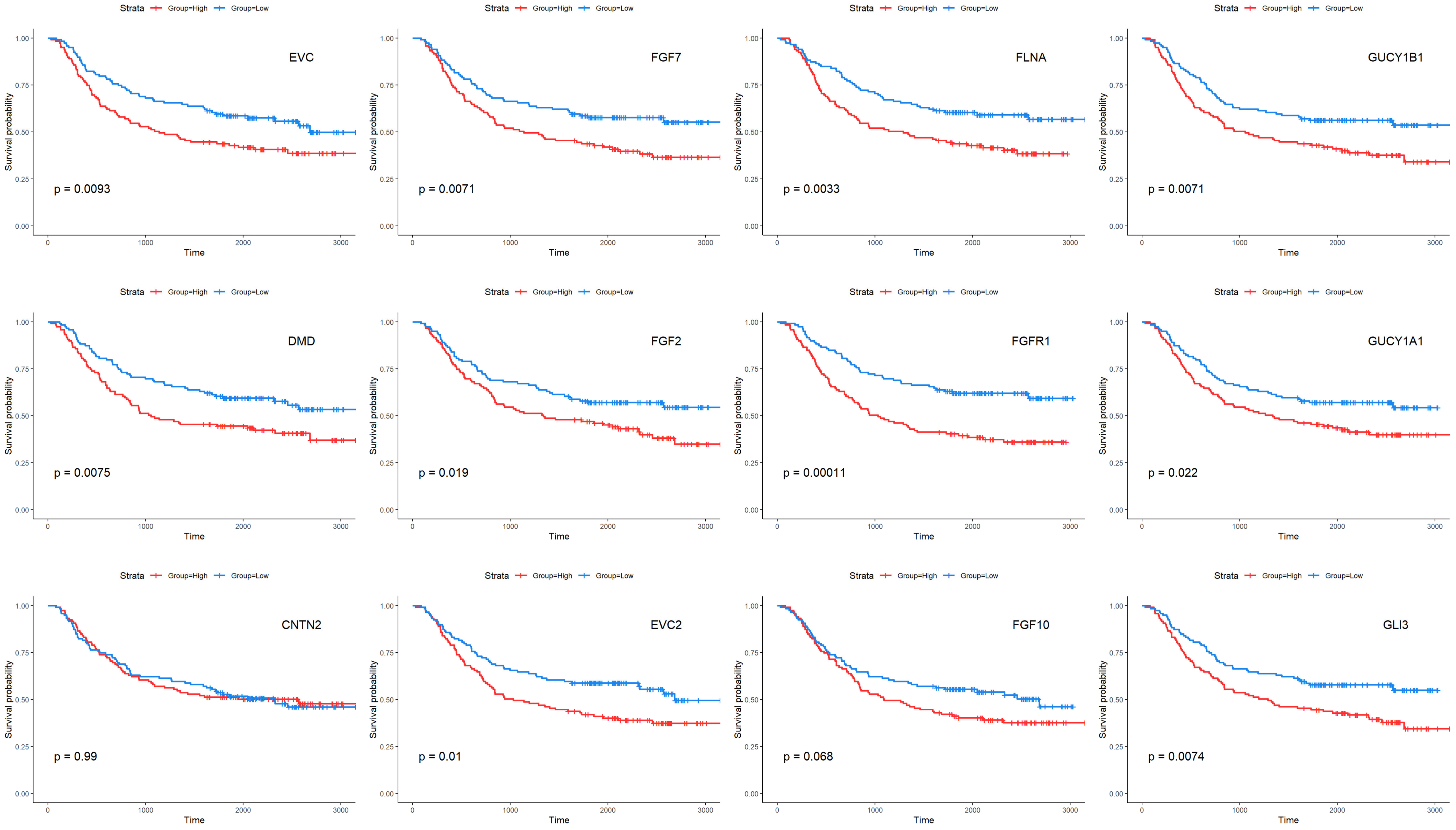
EVC
p = 0.0093
FGF7
p = 0.0071
FLNA
p = 0.0033
GUCY1B1
p = 0.0071
DMD
p = 0.0075
FGF2
p = 0.019
FGFR1
p = 0.00011
GUCY1A1
p = 0.022
CNTN2
p = 0.99
EVC2
p = 0.01
FGF10
p = 0.068
GLI3
p = 0.0074
Strata
Group=High
Group=Low
Survival probability
Time

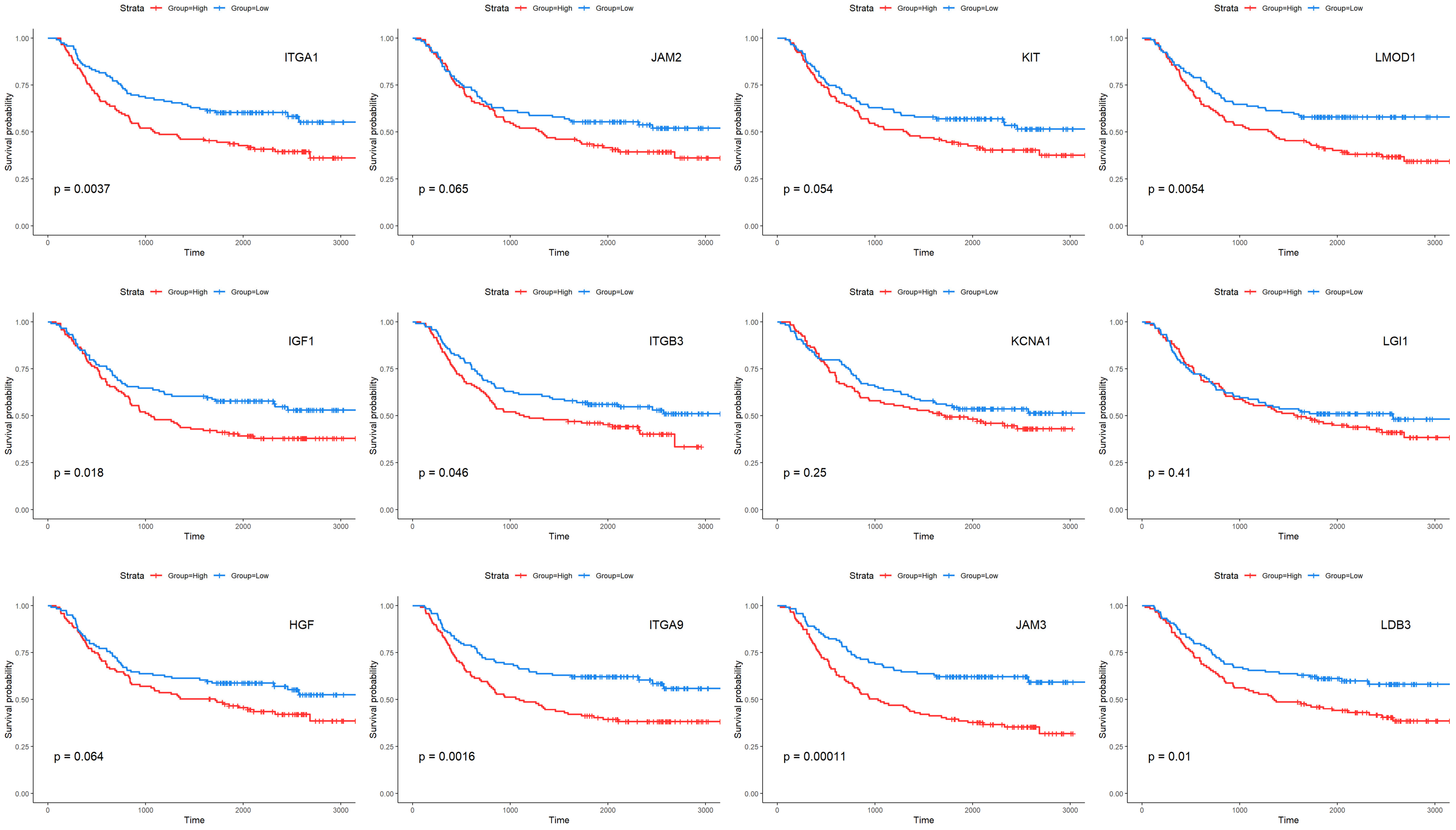
Strata Group=High Group=Low
Survival probability
Time
ITGA1
p = 0.0037
JAM2
p = 0.065
KIT
p = 0.054
LMOD1
p = 0.0054
IGF1
p = 0.018
ITGB3
p = 0.046
KCNA1
p = 0.25
LGI1
p = 0.41
HGF
p = 0.064
ITGA9
p = 0.0016
JAM3
p = 0.00011
LDB3
p = 0.01

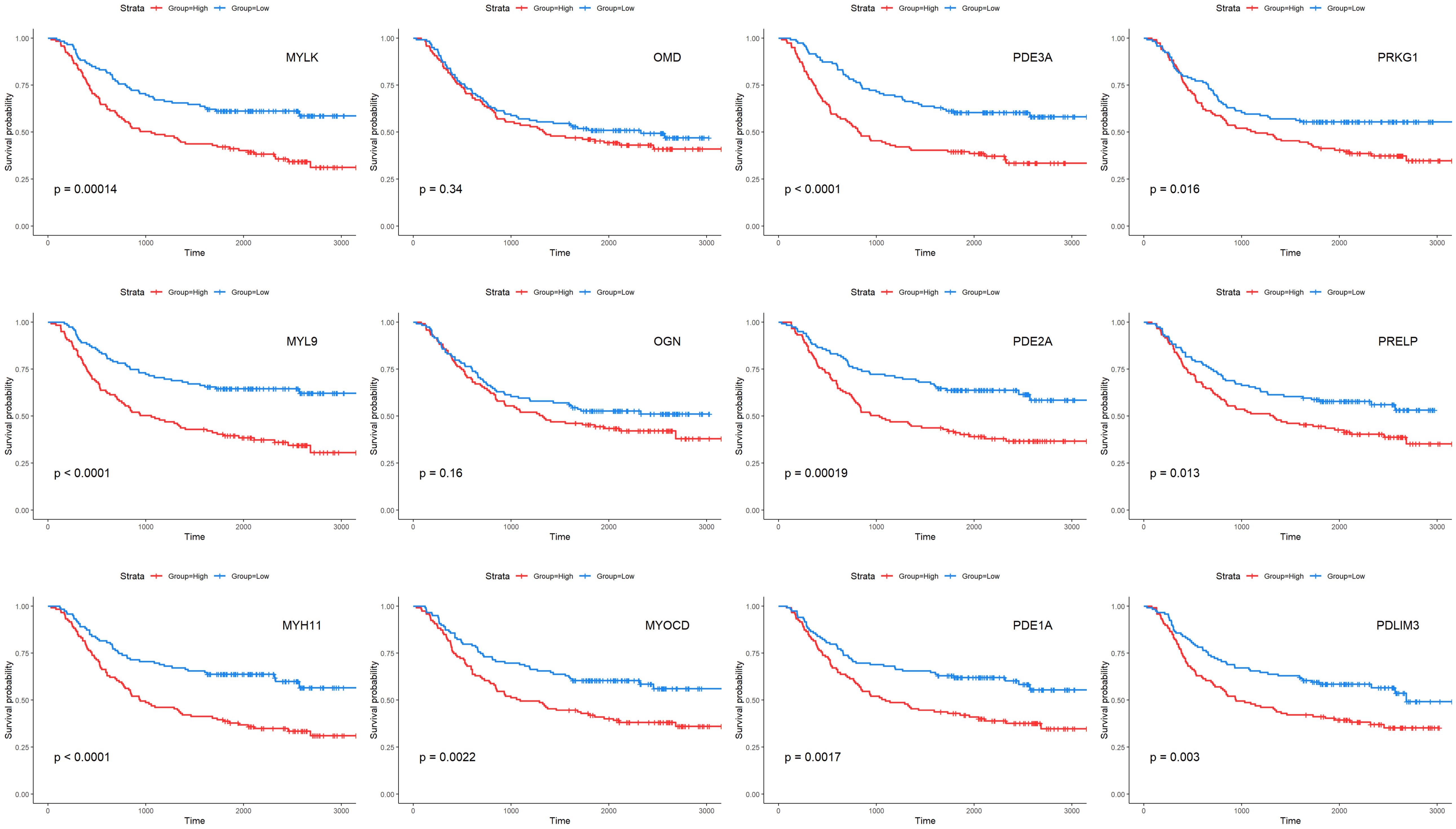
Strata
Group=High
Group=Low
MYLK
p = 0.00014
OMD
p = 0.34
PDE3A
p < 0.0001
PRKG1
p = 0.016
MYL9
p < 0.0001
OGN
p = 0.16
PDE2A
p = 0.00019
PRELP
p = 0.013
MYH11
p < 0.0001
MYOCD
p = 0.0022
PDE1A
p = 0.0017
PDLIM3
p = 0.003
Survival probability
Time
1.00
0.75
0.50
0.25
0.00
0
1000
2000
3000

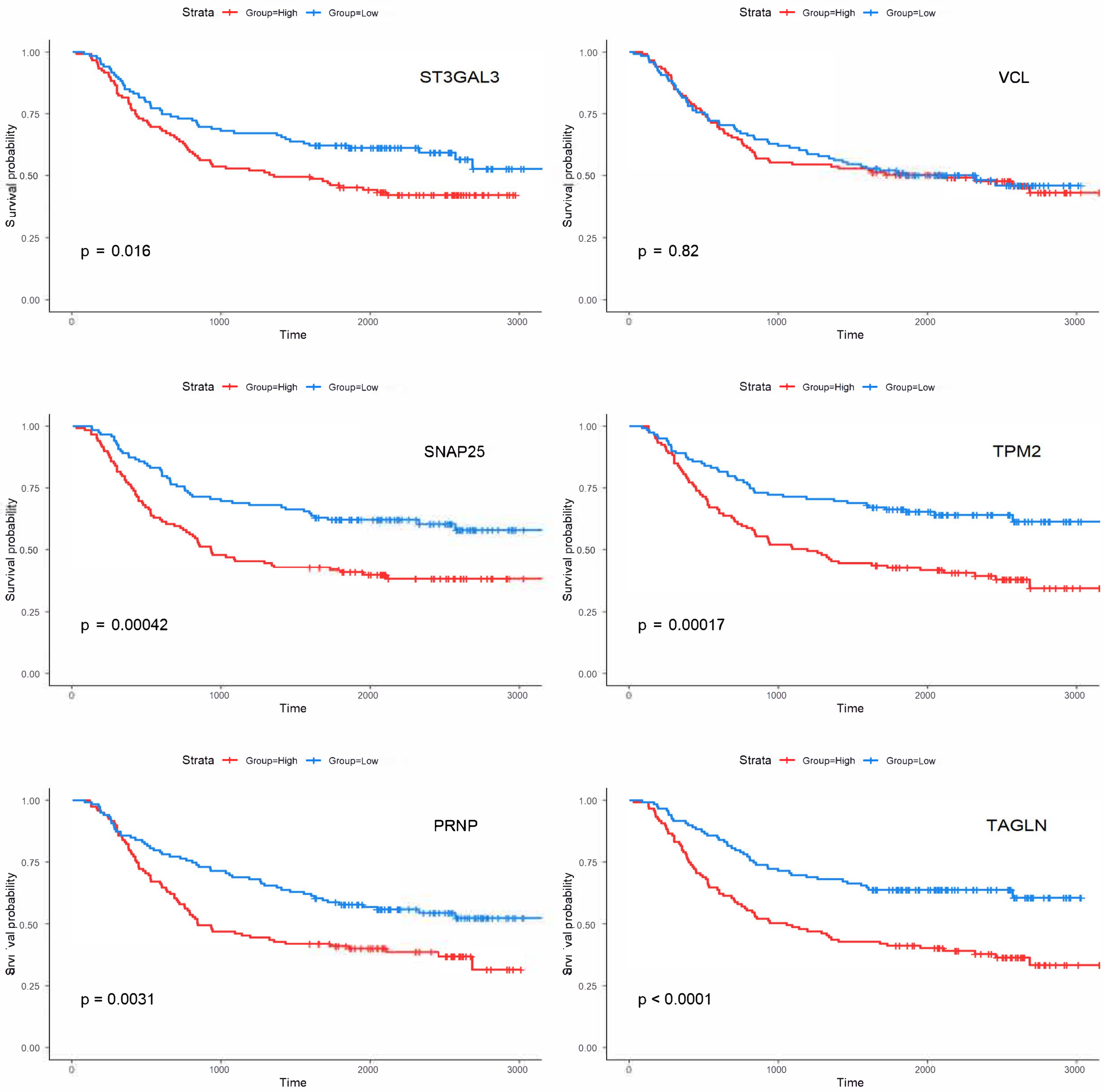

Supplementary Figure S5 Kaplan-Meier survival curves of the ACRG cohort for all of the top-scoring three-membered motifs as well as each member of the motifs in isolation. For motif members, the 'High' and Low' groups represent the top and bottom 40% of the' samples in terms of the expression of the respective genes. For the whole motifs, the intersection of the samples among the top 40% of samples and the bottom 40% of samples based on the expression of each of the three members of the respective motifs was used to form 'High' and 'Low' groups

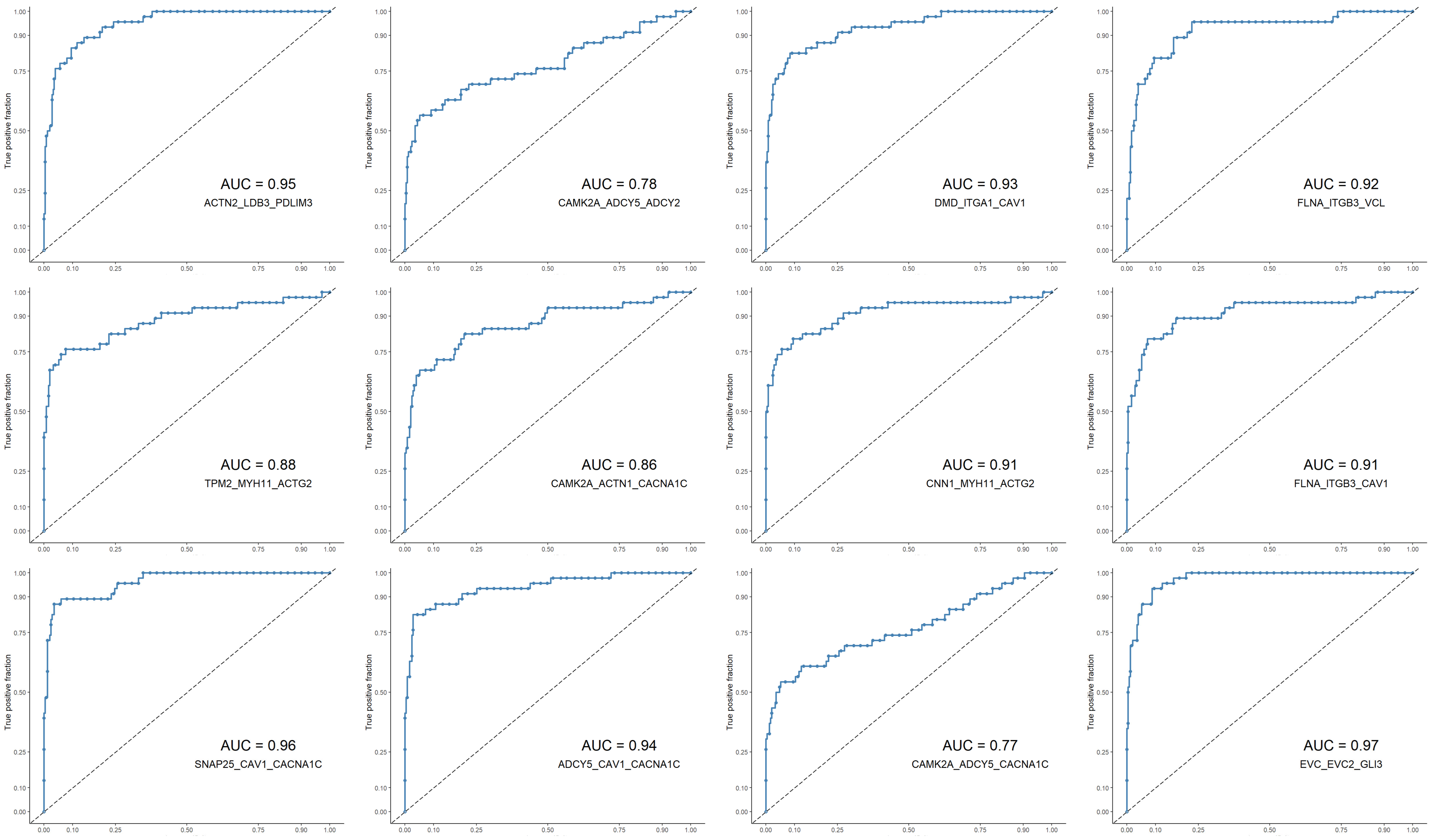
True positive fraction
AUC = 0.95
ACTN2_LDB3_PDLIM3
AUC = 0.78
CAMK2A_ADCY5_ADCY2
AUC = 0.93
DMD_ITGA1_CAV1
AUC = 0.92
FLNA_ITGB3_VCL
AUC = 0.88
TPM2_MYH11_ACTG2
AUC = 0.86
CAMK2A_ACTN1_CACNA1C
AUC = 0.91
CNN1_MYH11_ACTG2
AUC = 0.91
FLNA_ITGB3_CAV1
AUC = 0.96
SNAP25_CAV1_CACNA1C
AUC = 0.94
ADCY5_CAV1_CACNA1C
AUC = 0.77
CAMK2A_ADCY5_CACNA1C
AUC = 0.97
EVC_EVC2_GLI3
0.00
0.10
0.25
0.50
0.75
0.90
1.00

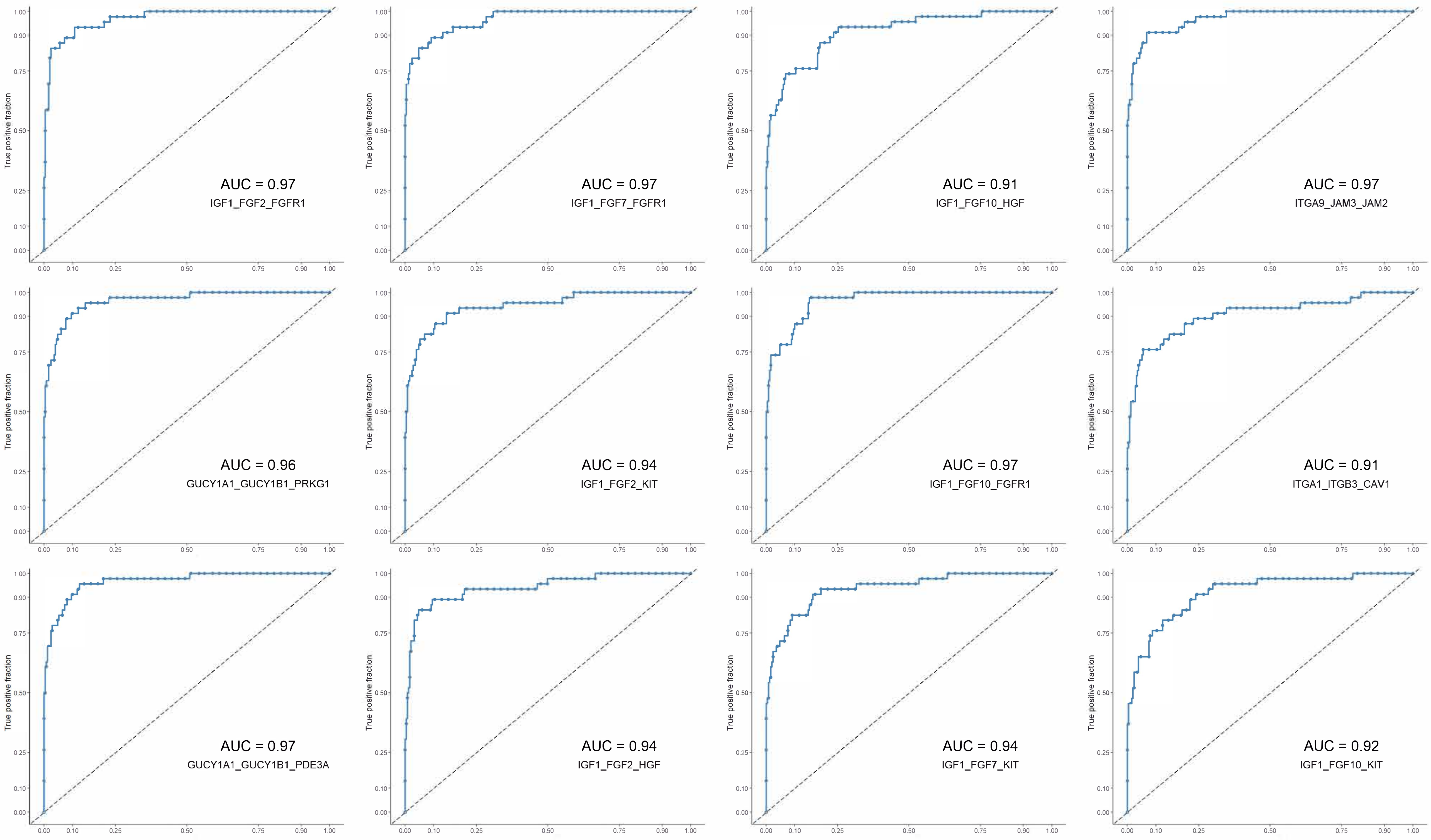
True positive fraction
AUC = 0.97
IGF1_FGF2_FGFR1
AUC = 0.97
IGF1_FGF7_FGFR1
AUC = 0.91
IGF1_FGF10_HGF
AUC = 0.97
ITGA9_JAM3_JAM2
AUC = 0.96
GUCY1A1_GUCY1B1_PRKG1
AUC = 0.94
IGF1_FGF2_KIT
AUC = 0.97
IGF1_FGF10_FGFR1
AUC = 0.91
ITGA1_ITGB3_CAV1
AUC = 0.97
GUCY1A1_GUCY1B1_PDE3A
AUC = 0.94
IGF1_FGF2_HGF
AUC = 0.94
IGF1_FGF7_KIT
AUC = 0.92
IGF1_FGF10_KIT

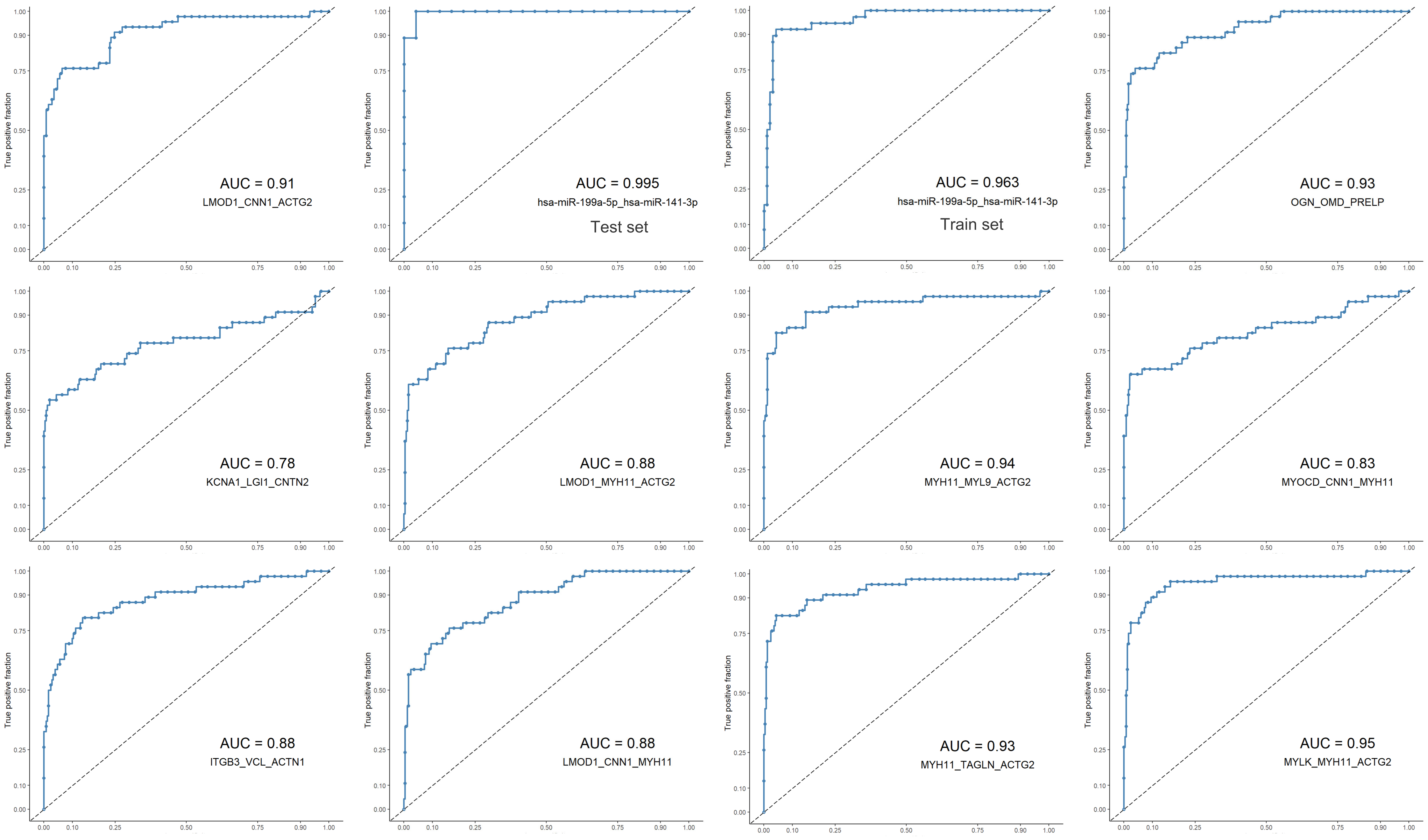

True positive fraction
AUC = 0.91
LMOD1_CNN1_ACTG2
AUC = 0.995
hsa-miR-199a-5p_hsa-miR-141-3p
Test set
AUC = 0.963
hsa-miR-199a-5p_hsa-miR-141-3p
Train set
AUC = 0.93
OGN_OMD_PRELP
AUC = 0.78
KCNA1_LGI1_CNTN2
AUC = 0.88
LMOD1_MYH11_ACTG2
AUC = 0.94
MYH11_MYL9_ACTG2
AUC = 0.83
MYOCD_CNN1_MYH11
AUC = 0.88
ITGB3_VCL_ACTN1
AUC = 0.88
LMOD1_CNN1_MYH11
AUC = 0.93
MYH11_TAGLN_ACTG2
AUC = 0.95
MYLK_MYH11_ACTG2

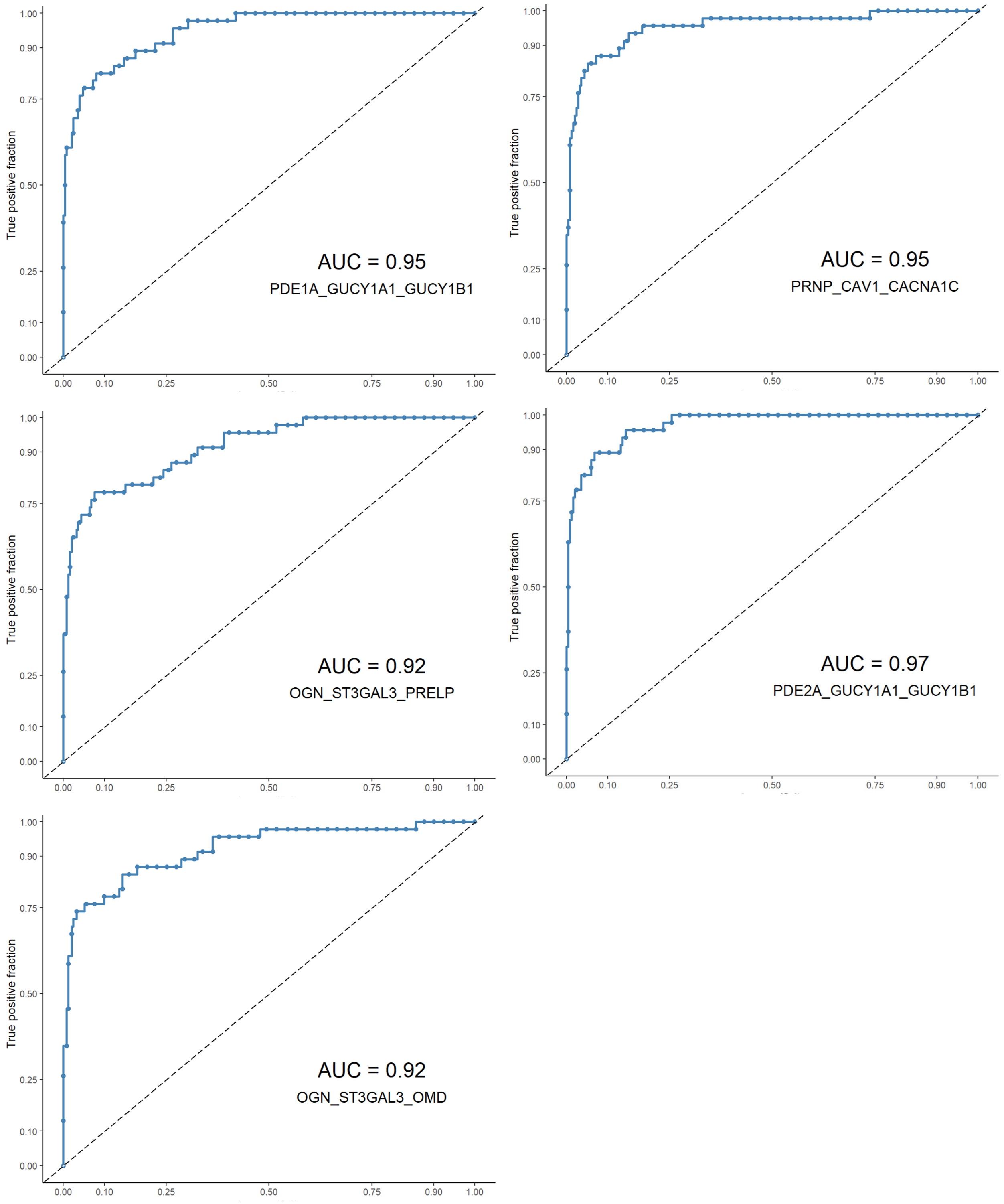

Supplementary Figure S6 The receiver operating characteristic (ROC) curves of the diagnostic performance of the models trained on TCGA cohort data in the validation cohort. The ACRG cohort was used for validation of the diagnostic performance of the mRNA motifs. In addition, the diagnostic performance of the miRNA-based discriminating model in both training (80% of TCGA samples) and test (the remaining TCGA samples) sets is also shown
