# Supplementary material for: Network-Based and Machine-Learning Approaches Identify Diagnostic and Prognostic Models for EMT-Type Gastric Tumors
Source: Genes (Basel). 2023 Mar 19;14(3):750. doi: 10.3390/genes14030750 (PMC10048224; doi:10.3390/genes14030750)
Supplement: Supplementary file 1 [file genes-14-00750-s001.zip › Supplementary Figure S2.pdf]

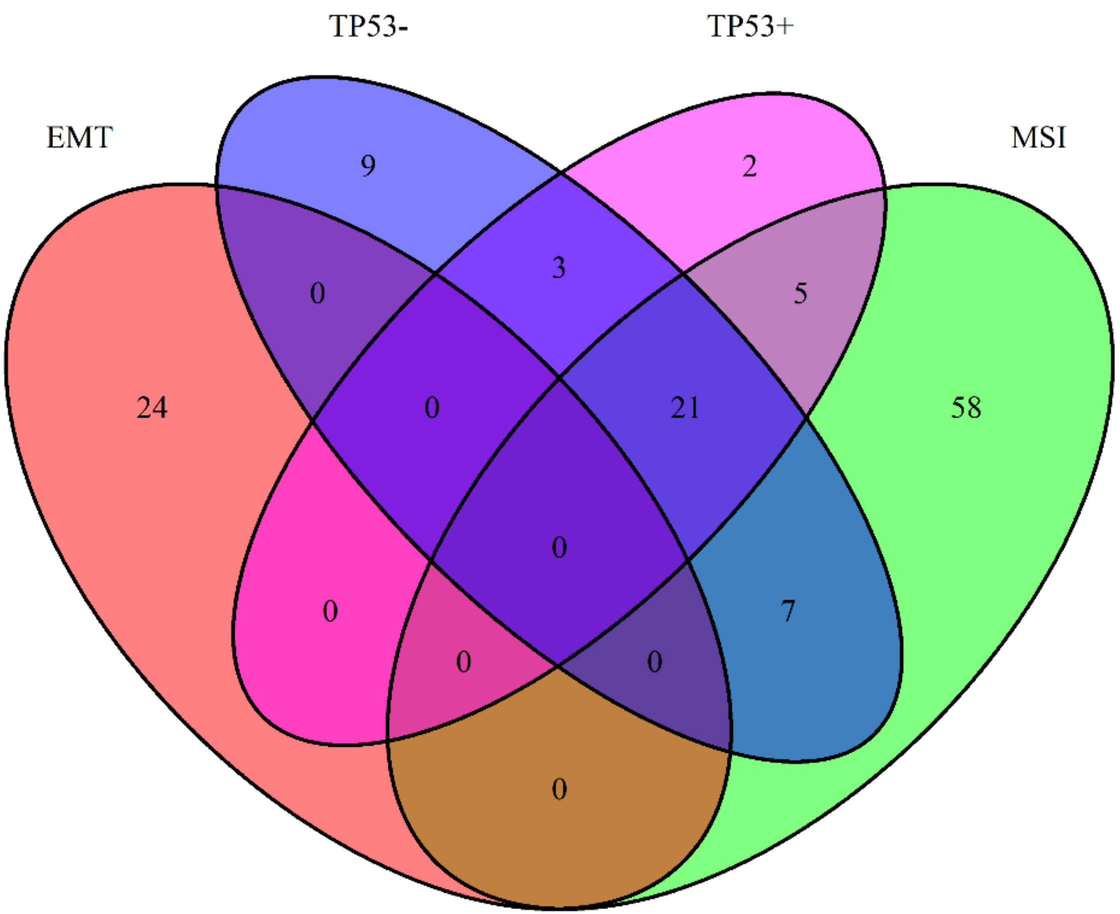

Supplementary Figure S2 The Venn diagram of the overlap of the members of the top protein-protein interaction modules of different ACRG gastric cancer subtypes. These modules were identified using the jActiveModules plug-in of the Cytoscape. Notice that while there are considerable overlaps between the members of the top modules of the MSI, TP53+, and TP53- subtypes, the top module of the EMT subtype shares no members with other modules
